# Aerosol drug delivery to spontaneously-breathing preterm neonates: lessons learned

**DOI:** 10.1186/s12931-020-01585-9

**Published:** 2021-02-26

**Authors:** Federico Bianco, Fabrizio Salomone, Ilaria Milesi, Xabier Murgia, Sauro Bonelli, Elena Pasini, Raffaele Dellacà, Maria Luisa Ventura, Jane Pillow

**Affiliations:** 1 Department of Preclinical Pharmacology, R&D, Chiesi Farmaceutici S.P.A., 43122 Parma, Italy; 2 Scientific Consultant, 48640 Bilbao, Spain; 3 TechRes Lab, Dipartimento Di Elettronica, Informazione E Bioingegneria (DEIB), Politecnico Di Milano University, Milano, Italy; 4 Neonatal Intensive Care Unit, Fondazione MBBM-ASST-Monza, Monza, Italy; 5 School of Human Sciences, University of Western Australia, Perth, Australia

**Keywords:** Aerosol delivery, Non-invasive ventilation, Nebulizer, Pulmonary drug delivery, Premature infants, Surfactant, Respiratory distress syndrome

## Abstract

Delivery of medications to preterm neonates receiving non-invasive ventilation (NIV) represents one of the most challenging scenarios for aerosol medicine. This challenge is highlighted by the undersized anatomy and the complex (patho)physiological characteristics of the lungs in such infants. Key physiological restraints include low lung volumes, low compliance, and irregular respiratory rates, which significantly reduce lung deposition. Such factors are inherent to premature birth and thus can be regarded to as the *intrinsic factors* that affect lung deposition. However, there are a number of *extrinsic factors* that also impact lung deposition: such factors include the choice of aerosol generator and its configuration within the ventilation circuit, the drug formulation, the aerosol particle size distribution, the choice of NIV type, and the patient interface between the delivery system and the patient. Together, these extrinsic factors provide an opportunity to optimize the lung deposition of therapeutic aerosols and, ultimately, the efficacy of the therapy.

In this review, we first provide a comprehensive characterization of both the intrinsic and extrinsic factors affecting lung deposition in premature infants, followed by a revision of the clinical attempts to deliver therapeutic aerosols to premature neonates during NIV, which are almost exclusively related to the non-invasive delivery of surfactant aerosols. In this review, we provide clues to the interpretation of existing experimental and clinical data on neonatal aerosol delivery and we also describe a frame of measurable variables and available tools, including in vitro and in vivo models, that should be considered when developing a drug for inhalation in this important but under-served patient population.

## Background

Premature infants are a heterogeneous patient population comprising infants of different gestational age, weight, and organ development; these factors contribute to a plethora of clinical conditions, including respiratory diseases [1]. Infants born at an early GA have structurally and functionally immature lungs and can be classified according to their ability to breathe spontaneously, which is a major determinant of the level of the required respiratory support. Spontaneous breathing depends on not only lung maturation, but also on the developmental status of the central nervous system and its ability to manage the respiratory drive and the control of respiratory muscles [2, 3].

The prompt identification of patients who can breathe independently is particularly important, given the widely established detrimental effects of invasive mechanical ventilation on the premature lung [4]. Premature infants exposed to mechanical ventilation are at higher risk of developing bronchopulmonary dysplasia (BPD) [5] a chronic lung disease that is preceded by pulmonary inflammation and which ultimately leads to abnormal lung development [6–8]. Indeed, mechanical ventilation should be restricted to those infants with inadequate respiratory drive, to reduce the incidence of detrimental long-term pulmonary consequences [9]. Premature infants may also eventually develop one or more associated pulmonary comorbidities such as postnatal pulmonary infections [10] or persistent pulmonary vascular hypertension [11].

Several techniques to increase the success of non-invasive ventilation (NIV) were developed over the last twenty years for the management of neonatal Respiratory Distress Syndrome (nRDS) [12]. The clinical efficacy of these techniques and associated reduced exposure of the fragile immature lungs to deleterious mechanical ventilation contributed to a gradual switch in the management of spontaneously-breathing infants. Consequently, NIV instead of mechanical ventilation is increasingly the standard of care [9].

In addition to NIV, spontaneously-breathing premature infants still require pharmacological interventions to treat the comorbidities associated with lung immaturity. These  treatments may include intravenous antibiotics [13], corticosteroids [14], and intratracheal exogenous surfactant [9, 15]. The lung bioavailability of these drugs could be increased significantly if these drugs were administered directly to the pulmonary site of action via aerosol delivery, thereby reducing the undesired systemic exposure of some medications [16]. Moreover, in the case of surfactant replacement therapy, aerosol delivery could mitigate the associated iatrogenic risks of intubation for intrapulmonary surfactant delivery, and sustain the beneficial effect of non-invasive respiratory support [17–19]. Moreover, the development of a method to deliver aerosolized drugs efficiently to premature infants could also give rise to new pharmacological therapies. Relevant therapies that show encouraging results in preclinical studies include anti-inflammatory drugs [20–25], anti-infectives [26, 27], molecules promoting epithelial growth and integrity (e.g. Vitamin A and D) [28–31], vasodilators [32], as well as surfactants [19, 33–36].

Non-invasive pulmonary drug delivery to premature infants has been explored with several different devices either integrated within the NIV circuit (e.g. nebulizers) [37–39] or given to the patients during temporary cessation of NIV (e.g. using a pressurized metered dose inhaler with a spacer chamber) [40]. Nevertheless, although aerosol delivery is an intuitive concept as a targeted method to deliver medications during NIV, very low actual lung deposition rates (< 1% of the nominal dose) are reported in the literature for premature infants [39, 41]. Further, no aerosolized drug is approved for this patient population. Consequently, aerosol delivery to spontaneously-breathing premature infants managed with NIV remains a significant unmet clinical need.

The challenges of developing a drug for nebulization in premature infants are highlighted by the undersized anatomy and the complex (patho)physiological characteristics of the lungs in such infants. Key physiological restraints include low lung volumes, low compliance, and heterogeneous respiratory rates. These characteristics reduce lung deposition of aerosolized medications compared to older paediatric patients and adults [42, 43]. Such factors are inherent to premature birth and thus we will refer to them as the *intrinsic factors* that affect lung deposition. However, *extrinsic factors* also impact deposition: such factors include the choice of aerosol generator and its configuration within the ventilation circuit, the drug formulation, the aerosol particle size distribution, the choice of NIV type, and the patient interface between the delivery system and the patient. Together, these extrinsic factors provide an opportunity to optimize the lung deposition of therapeutic aerosols and, ultimately, the efficacy of the therapy.

This review addresses the key variables that influence the efficacy of drug aerosolization in spontaneously-breathing preterm infants managed with NIV, and consider both the intrinsic and extrinsic factors that may affect the delivered lung dose. We will also consider preclinical and clinical evidence published to date. Our aim is to provide clues to the interpretation of existing data and to describe a frame of measurable variables and available tools, including in vitro and in vivo models, that should be considered when developing a drug for inhalation in this important but under-served patient population.

## Factors affecting lung deposition in preterm neonates

Delivery of medications to preterm neonates managed with NIV represents one of the most challenging scenarios for aerosol medicine. The process encompassing aerosol generation and deposition is mainly governed by the laws of fluid dynamics. Therefore, the aerosol particle size distribution, the air-flows within the NIV circuit and in pulmonary airways, and the calibre of the “pipes” through which the aerosol-containing air is transported decide the fate of therapeutic particles. Awareness of all the factors that influence aerosol deposition may give rise to strategies that maximize the efficiency of aerosol therapies directed to spontaneously-breathing premature neonates. Thus, in this section we address both the intrinsic and extrinsic factors that influence lung deposition during aerosol delivery to premature infants managed with NIV.

### Intrinsic factors affecting lung deposition

Lung deposition reported for mechanically-ventilated and spontaneously-breathing adults ranges between 5–10% and 10–25% of the nominal dose, respectively [43]. In contrast, the lung deposition values reported for both intubated and non-intubated premature neonates are very low at around 1% of the nominal dose [39, 41, 44]. This difference in lung deposition as a function of patient population is explained primarily by the smaller size of premature infants. However, other factors such as the anatomical and (patho)physiological differences between these patient groups account for the attenuated aerosol lung deposition in premature infants compared to adults.

#### Anatomy of the respiratory system of premature infants

**Fig. 1:**
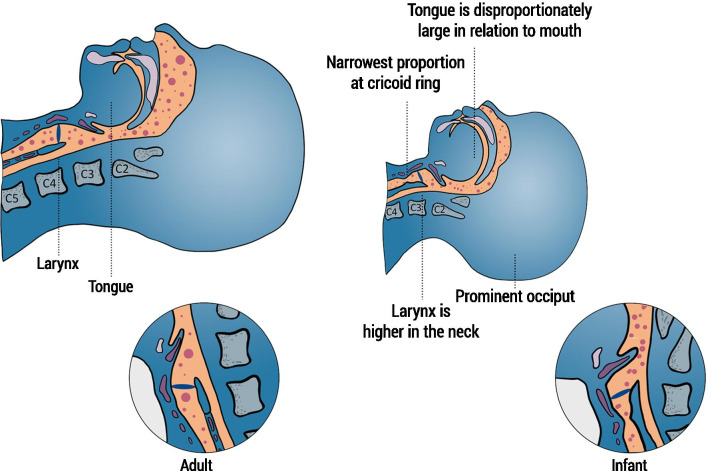
Differences in the anatomy of the pharynx and larynx of an adult (left) and an infant (right) and their effect on the pathway of aerosol particles (represented by pink dots). Premature infants receive nebulization while lying in a cot or in an incubator (upper panel) whereas nebulization is normally administered to adults with the patient in a seated or erect position (lower panel). Thus, the anatomical differences between adult and neonate are compared for a lying down position in Fig. 1. This graphic highlights the main differences that make the pathway of aerosol particles more curvy in premature infants, potentially reducing the effective delivery of nebulized substance to the lungs.

There are significant differences between the upper airway of newborn infants and adults. Compared with adult anatomy, the epiglottis is relatively high in the pharynx of newborn infants and therefore closer to the soft palate, which reduces the resistance to air entering through the nasopharynx (Fig. 1) [45]. Consequently, newborn infants are preferential nasal breathers until approximately five months of age [46]. For this reason, the attempts to deliver an aerosolized drug to infants have been almost exclusively performed through the nasal route. Aerosolization through the mouth may elicit the swallowing reflex as well as glottic closure promoting pharyngeal drug accumulation such that drug may subsequently be swallowed rather than inhaled. The narrow nasal passages account for up to 50% of airway resistance [47], and may therefore promote the impaction of therapeutic aerosol particles. The cross-sectional area of the nasal passages may decrease further due to endogenous secretions [48], or due to accumulation of nebulized aerosol particles. Another important anatomical difference between adults and newborns is the alignment of the epiglottis with the trachea: the epiglottis in adults is more aligned with the trachea compared with newborns [49]. In newborns, the epiglottis forms a more oblique angle with the trachea and imposes an additional impediment for the transport of aerosol particles from the pharynx into the trachea. Every angle encountered by the aerosol-containing air stream enroute to the peripheral lungs (e.g. branching angles of the *bronchi* and *bronchioles*) leads to significant inertial impaction of aerosol particles [50], in turn, reducing the amount of aerosol reaching the lung periphery. A fraction of the aerosols impacting on the airways may then be transported out of the lungs by the mucociliary clearance and swallowed [51].

The diameter of the trachea, bronchi and bronchioles are narrower in term newborns than in older children and adults. The estimated geometry of the neonatal trachea (Generation 0, G0) at term is characterized by a diameter of 5.4 mm and a length of 36 mm [52]. The cross-sectional area and length of the airways decrease progressively as the air-flow moves distally towards deeper airway generations; airway diameter at G10 is estimated to be 0.33 mm and it tapers down to 0.12 mm at G20 [52].

Airway calibre in premature neonates is even narrower; Fishman and Pashley analysed the airway size from specimens of 39 prematurely-born infants ranging between 21–40 weeks’ gestation and a birthweight ranging between 390–3 600 g [53]. They described a hyperbolic function relating tracheal diameter and birthweight: premature infants with a birthweight between 390 g and 1 000 g had a tracheal diameter ranging between 2.0–3.5 mm, whereas those with a birthweight between 1 000 g and 2 000 g had a tracheal diameter ranging between 3.5–4.0 mm. Whilst, infants with a birthweight higher than 2 000 g had a tracheal diameter ~ 4.0 mm. Similar tracheal dimensions were reported by Richards and Farah after visualizing the fetal upper airway of 120 patients by ultrasound imaging [54]. This sonographic study reported that the tracheal diameter increased from a mean of 2.4 mm at 18 weeks to 4.6 mm at 38 weeks. These narrow conducting airways favour the impaction of aerosol particles and promote a more central pattern of lung drug deposition [39].

#### (Patho)Physiology of the premature lung

The lungs of premature infants are structurally and biochemically immature. Most premature infants requiring ventilation support are born in the saccular phase of lung development, which spans from the 24th to the 36th week of gestation [55]. During the saccular phase, the alveolar ducts (defined as the last airway generation before the development of mature alveoli) start forming in the distal lung [55]. Also, the synthesis and secretion of pulmonary surfactant by the type II alveolar cells starts approximately at the 24th week of gestation and increases steadily until birth [56]. Surfactant in the alveolar space modulates the surface tension throughout the respiratory cycle, reducing it almost to zero at low gas volumes, preventing alveolar collapse at end-expiration [57]. Given the gradual increase in the intrapulmonary surfactant pools with advancing maturation, it follows that lowest levels of intrapulmonary surfactant will be in the most immature infants. Therefore, the most immature infants are at highest risk of developing nRDS [58]. Moreover, these younger infants are potentially also those with a higher requirement for less-invasive surfactant delivery via aerosol.

One of the main pathophysiological features of nRDS is a low distending lung volume at end tidal respiration, referred to as the functional residual capacity (FRC). The low FRC in infants with nRDS is a consequence of the low intrapulmonary surfactant pools at birth [59], as well as increased compliance of the chest wall of premature infants compared to the adult chest wall [60]. Consequently, the lungs of nRDS infants are characterized by widespread atelectasis, reduced pulmonary compliance, and low tidal volumes (*V*_T_) for which they try to compensate increasing the respiratory rate. Dekker et al*.* determined the tidal volume (*V*_T_) of 23 premature infants (GA 29^+0^; 27^+5^–31^+0^; mean and interquartile range, IQR) with a mean birth weight of 1 252 g (IQR 1 050–1 388 g) immediately after birth [61]; infants showed a mean *V*_T_ of 2.7 mL/kg (IQR 1.0–5.7 mL/kg) in the first 4 min of life, which increased to 2.9 mL/kg (IQR 1.3–5.4 mL/kg) in the first 7 min of life. The mean respiratory rate of the group of infants in the Dekker study was 23 breath/min (IQR 7–36 breath/min). te Pas et al*.* also investigated the first breaths immediately after birth of 13 term (birth weight 3340 ± 530 g) and 12 premature newborns (≥ 31 weeks’ GA, birth weight 2000 ± 560 g) who were not expected to require respiratory support [62]. They reported that 80% and 85% of the analysed breaths from premature and term newborns, respectively, showed braked expirations (i.e. expiratory hold postponing the main expiratory flow). The reported mean respiratory rate for premature infants with a breathing pattern with braked inspirations was 60 ± 30 breaths/min, with inspiratory and expiratory times of 0.32 ± 0.14 s and 1.03 ± 0.84 s, respectively. The mean respiratory rate during breathing with unbraked expirations was 90 ± 26 breaths/min with no difference in the inspiratory time (0.30 ± 0.13 s) compared with the braked breathing pattern but a significant shorter expiratory time (0.41 ± 0.16 s). The authors reported four braking mechanisms: prolonged expiration, breath hold, grunting, and crying [62]. Premature infants suffering from mild-to-moderate nRDS may display higher respiratory rate (> 60 breaths/min) and additional abnormal respiratory patterns, including periodic breathing and episodes of apnoea. Indeed, the frequency of apnoeic episodes correlates inversely with gestation, and apnoeas are present in nearly all infants born at less than 26 weeks’ gestation [63].

Compared to other paediatric populations or adults, the tidal breathing indices of premature infants set a challenging scenario for aerosol medicine (Table 1). Low *V*_T_ and high respiratory rate with short inspiratory times combine to limit the amount of aerosol entering the lungs of premature infants and reduce the residence time of aerosol particles within the lungs, which has a significant negative impact on lung deposition.

**Table 1:** Representative respiratory indices across the lifespan

	Premature newborn infant (2 kg)*	Healthy newborn (10 d old)	7-month-old	5-year-old	Adult (53-year-old)
RR, cycles/min	90	44	25	21	12
I:E	1:2.5	1:3	1:2	1:2	1:2
T_i_, s	0.30	0.34	0.80	0.95	1.67
Flow, L/min	2.9	3.8	6.5	11.2	18.0
*V*_T_, mL	7.4	22.0	87.0	177.0	500.0
% of adult *V*_T_	1.5	4.4	17.4	35.4	100
*V*_E_, L	0.66	0.96	2.17	3.71	6.00

Adapted from Xi et al.[64]

RR, respiratory rate; I:E, Inspiratory-expiratory ratio; T_i_, inspiratory time; *V*_T_, tidal volume; *V*_E_, minute ventilation

*Premature infant data from te Pas et al. [62]; representative data from other pediatric populations and adults from Fleming et al., Gagliardi et al., and Rusconi et al.[65–67]

#### Practical example of aerosol deposition in a spontaneously-breathing premature infants

**Fig. 2:**
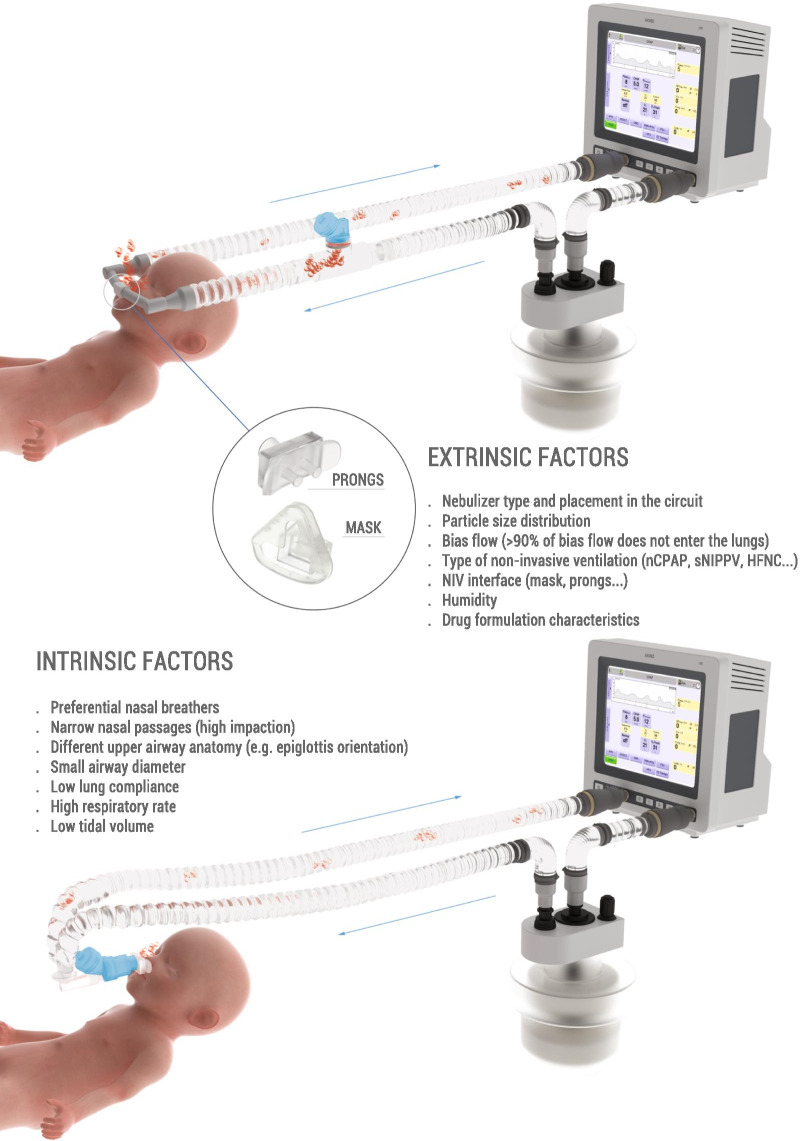
Intrinsic and extrinsic factors influencing aerosol drug delivery in premature infants. The figure depicts two possible scenarios: (1) nebulizer positioned in the inspiratory limb of a standard constant flow ventilator (top); this nebulizer positioning could also be applied to bubble Continuous Positive Airway Pressure (CPAP) and variable flow drivers, and to High Flow Nasal Cannula (HFNC); (2) nebulizer positioned between the Y piece and the patient interface in a standard constant flow ventilator (bottom); this nebulizer positioning could be compatible with bubble CPAP but not with variable flow drivers nor HFNC

The low efficiency of nebulized therapies in premature infants is illustrated with a simplified example. Imagine a premature infant (~ 29 weeks’ gestation, ~ 1 250 g birthweight) supported with nasal Continuous Positive Airway Pressure (nCPAP) delivered by a ventilator. The clinicians aim to treat this infant with a nominal dose of 2.0 mg/kg of a drug administered via nebulization with the nebulizer positioned within the inspiratory limb of the nCPAP circuit, as in Fig. 2 (top). If the bias flow is 7 L/min and the *V*_T_ and respiratory rate of this premature infant are 6.0 mL/kg and 60 breaths/min, respectively, these respiratory parameters would yield a minute ventilation (*V*_E_) of 0.45 L/min. If we assume that the nebulizer has a constant aerosol drug output of 2.5 mg/min, then the aerosol particles will homogeneously distribute in the 7 L of gas that feeds the inspiratory limb every minute, reaching an aerosol drug concentration of 0.35 mg/L in the inspired gas. Under these conditions, the maximum lung dose in this example would be 0.15 mg (or 0.12 mg/kg), accounting for just 6% of the nominal dose. However, in a real clinical scenario the maximum dose would be diminished further by: (1) the residual drug remaining in the nebulizer; (2) the amount of aerosol depositing in the NIV circuit and in the nCPAP interface; and (3) the aerosol that enters the lung but is rapidly exhaled due to the low lung residence time of the particles within the lungs [42]. If a jet nebulizer was used, the aerosol would be diluted (and hence inhaled dose reduced) even further by the driving gas required to generate the aerosol (e.g. 2–6 L/min for low gas volume jet nebulizers) that would decrease the aerosol drug concentration in the inspiratory air-flow to less than 0.71 mg/L. This example assumes a constant and regular respiratory rate and does not consider potential aerosol losses due to air-leaks, which are common during NIV and will further compromise aerosolized drug delivery.

In summary, the patient-related, intrinsic factors of spontaneously-breathing premature infants set a significantly challenging situation for aerosol medicine. Nevertheless, gaining awareness of the anatomy and physiology of this patient population is essential to implement neonate-focused aerosol delivery strategies that will further optimize lung deposition. Therefore, the next section describes the factors that are extrinsic to premature infants (e.g., NIV type, nebulizer, patient interface) and which influence aerosol deposition and, ultimately, also the delivered lung dose.

### Extrinsic factors affecting lung deposition

#### Aerosol delivery devices

Nebulizers, pressurized metered dose inhalers (pMDI), and dry powder inhalers (DPI) are the most common devices used in aerosol medicine [68]. pMDIs use a propellant under pressure to generate a metered aerosol dose through a nozzle and they are widely used in the daily management of Asthma and Chronic Obstructive Pulmonary Disease (COPD). DPIs deliver a dose of a drug as a powder along with the inspiratory effort of the patient [69]. Inspiratory flows > 30 L/min are usually required to generate aerosols with DPIs and therefore are not suited to be used in preterm neonates. Moreover, to optimize pulmonary deposition, pMDI require breath coordination with device actuation, which are not achievable in premature infants. Nevertheless, pMDIs can be used in neonates in combination with a spacer chamber that creates an aerosol reservoir from which the aerosol drug particles can be inhaled. pMDIs with spacer chambers have been used for the treatment of premature infants with BPD [40, 41]. Indeed, they can be included into ventilation circuits or can be used with spontaneously-breathing infants temporarily disconnected from NIV.

Nebulizers can be classified as ultrasonic, jet, and vibrating-membrane devices [68]. Ultrasonic nebulizers use a rapidly vibrating pressure-responsive electric crystal that transmits the vibrations through the liquid medication to its surface, creating aerosol droplets from the crests of the surface waves [70]. Ultrasonic nebulizers are relatively expensive, may heat the medication, and show attenuated efficiency with viscous formulations such as suspension [68]. These issues compromise the delivery of surfactant, which is a phospholipid suspension containing natural peptides that are denaturalized by increased temperature. Therefore, clinicians have used jet and vibrating-membrane nebulizers rather than ultrasonic nebulizers for prolonged aerosol therapy (usually for the administration of nebulized surfactant) in spontaneously-breathing premature infants managed with NIV [37, 38, 71–74]. Jet or pneumatic nebulizers use compressed gas to break up liquids into aerosols, usually at gas flows ranging between 4–6 L/min. Aerosol particle size and drug output can be altered, to a certain extent, by modifying the flow of the driving gas [70]. However, in vibrating-membrane nebulizers a membrane with 1000–7000 laser-drilled holes vibrates at the top of the liquid reservoir thereby generating a mist of very fine droplets out through the holes [75].

**Fig. 3:**
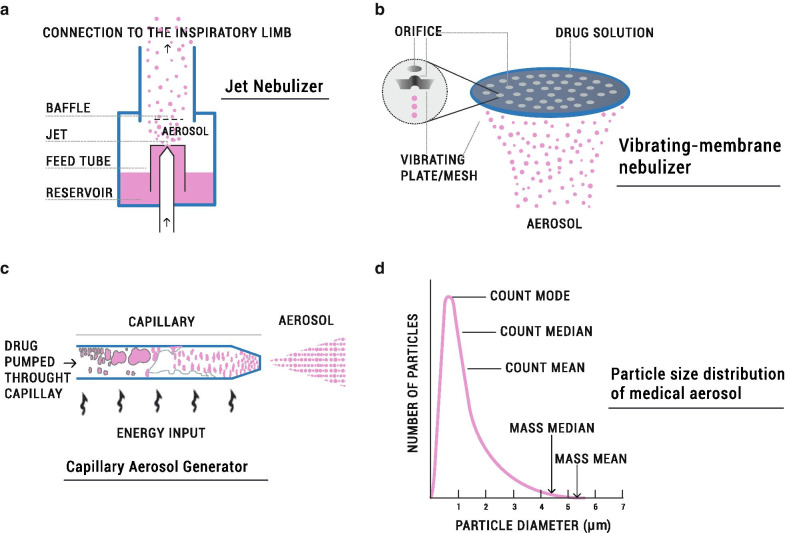
**a**–**c** Illustrate different types of aerosol generators used to deliver aerosols to spontaneously-breathing premature-infants. Jet or pneumatic nebulizers use compressed gas to break up liquids into aerosols and incorporate baffles to filter large aerosol particles (**a**). Vibrating-membrane nebulizers consist of a membrane with 1000–7000 laser-drilled holes that vibrate at the top of the liquid reservoir thereby generating a mist of very fine droplets through the holes (**b**). The capillary aerosol generator (CAG) has been especially designed to deliver synthetic surfactant aerosols; this technology consists of a heated capillary through which surfactant is pumped and further dispersed as an aerosol (**c**). Medical aerosols usually conform to a log-normal particle size distribution (**d**); They are usually defined by their Mass Median Aerodynamic Diameter (MMAD) which determines the particle diameter at which half of the aerosolized drug mass lies below and half above the stated diameter. Particle size distribution is usually given as the Mass Median Diameter (MMD), which is not interchangeable with the MMAD. MMD is the output parameter in laser-diffraction experiments and considers the particles to be spherical and of unit density. It should be noted that the MMAD and MMD appear markedly shifted to the right in the distribution compared with the particle diameter mode, median, and mean of the absolute particle counts. **a**–**c** adapted from reference [79] and **d **adapted from reference [70]

Jet nebulizers were the predominant nebulizer technology used for clinical investigations with aerosolized surfactant [37, 71, 72], until the emergence of the vibrating-membrane nebulizer technology two decades ago [38, 73]. Indeed, there is now substantial evidence from in vitro and in vivo studies showing that vibrating-membrane nebulizers outperform jet nebulizers when use in ventilated neonates [76, 77]. Dubus et al*.* compared the lung deposition of the model drug ^99m^Tc diethylenetriamine pentaacetate (^99m^Tc-DTPA) delivered with either a jet nebulizer (Misty-Neb) or a vibrating-membrane nebulizer (Aeroneb Professional) to macaques (2.6 kg). They reported a lung deposition of 0.5% of the nominal dose with the jet nebulizer, whereas lung deposition was as high as 14.0% with the vibrating-membrane nebulizer [77]. The superior performance of vibrating-membrane nebulizers in the context of neonatal care is attributed mainly to their low residual volume (i.e. the liquid volume that remains in the nebulizer after the end of a treatment) and the absence of added air-flow to the ventilation circuit. Further, aerosol particles exit the nebulizer at high velocity after jet nebulization: high velocity flows increase the extra-thoracic aerosol deposition due to inertial impaction within the NIV circuit, patient interface, and upper airways [78]. Conversely, vibrating-membrane nebulizers release slow, highly-concentrated aerosols that can be more readily delivered to the patient by the bias flow through the NIV circuit (Fig. 3).

In addition to jet and vibrating-membrane nebulizers, a specific drug/device combination, undergoing clinical evaluation (NCT02636868), uses a capillary aerosol generator (CAG) to produce KL4 synthetic surfactant aerosols (Fig. 3). CAG technology consists of a heated capillary through which surfactant is pumped and then dispersed as an aerosol. Interestingly, the aerosol plume produced with the CAG device displays a low velocity output.

*Inhalation* or *atomizing* catheters are proposed as a method to deliver surfactant during NIV [80–82]. Unlike most aerosol devices, which create droplets outside the patient, atomizing catheters are designed to maximize the dose delivered to the lung by generating intra-corporeal, yet extra-thoracic, aerosols. The design of atomizer devices includes long (30–120 cm) and very thin catheters made of at least two or more channels conveying pressurized gas and drug to the catheter tip, where the aerosol plume is produced [80, 83–85]. For aerosol delivery during NIV, inhalation catheters can be inserted into a patient-specific buccal interface that is designed to keep the catheter in place (i.e. with the tip of the catheter pointing to the vocal cords). Recently, a clinical trial compared the aerosol delivery of surfactant (*Calfactant*, Infasurf®) using such an inhalation catheter type (*Solarys* aerosol generator, Trudell Medical International), with “usual clinical care” in spontaneously-breathing nRDS patients (NCT03058666). The device is a modified Solarys aerosol generator consisting of an atomizing catheter positioned in the mouth of the infants and which is kept in place by a dedicated pacifier. The authors have reported a significantly lower rate of intubation for liquid surfactant instillation in the intervention group [86]. Unfortunately, no information regarding aerosol particle size distribution (APSD) or lung deposition was provided, which may raise questions on the claim that the clinical benefit observed in a subset of the population studied can be attributable to aerosolized surfactant without any doubt. A slightly different strategy of aerosolization was described in a series of late-stage preclinical investigations [81, 82]: an alternative atomizing catheter was kept in place by a custom-made interface placed in the retro-pharynx close to the vocal cords, fostering intra-tracheal deposition. This technology is effective in delivering surfactant (*Poractant alfa,* Curosurf®) to preterm lambs with nRDS managed with nCPAP [81]. Separate studies in healthy, newborn piglets using scintigraphy show remarkable lung deposition ranging between 24–68% of the nominal surfactant dose [82].

The delivery of surfactant as a dry powder is feasible in vivo using prototypes of aerosol generators. Walther et al*.* reported the delivery of synthetic dry powder surfactant aerosols to animal models of respiratory distress managed with NIV using a device designed by Acorda Therapeutics [36]. The device consists of a cylindrical, low-flow aerosolization chamber with one or more holes at one end that can accommodate a perforated capsule containing the surfactant powder. The powder is dispersed at low flows (4–10 L/min), and the device can be incorporated into a CPAP circuit [36]. Notably, dry powder surfactant particles tend to aggregate upon delivery [87]. Rahmel et al*.* also reported that such aggregation phenomena were associated with the formation of clots blocking the trachea of preterm lambs. To circumvent this serious safety issue, Pohlmann et al*.* developed a continuous powder aerosolizer (CPA) that includes a further aerosol humidification step before delivering the dry powder surfactant to the patient [88]. The device consists of a supply unit that provides pressure pulses to the dry powder surfactant container, a spacer chamber for the aerosol cloud to develop, and an additional container that provides humidification to the aerosol particles before they are delivered to the patient. This way, dry surfactant particles are covered with a surface layer of water that avoids aggregation.

#### Aerosol characteristics

The aerosol characteristics for each drug/device combination are defined by the active substance delivery rate, the total active substance delivered, and the APSD. Any investigational drug for aerosolization has to be characterized according to requirements set by the official compendia European Pharmacopeia [89] and US Pharmacopeia [90] to be approved by regulatory authorities, since the performance of the device during aerosolization can significantly impact drug distribution in the airways and ultimately its pharmacological activity. Both Pharmacopeia set three mandatory tests in order to characterize a preparation for nebulization:  (1) active substance delivery rate;  (2) total active substance delivered; and (3) aerodynamic assessment of the nebulized aerosol (APSD).

The active substance delivery rate and the total active substance delivered represent an estimation of the rate and amount of drug that reaches the patient. These parameters are determined collecting the drug after in vitro nebulization on a drug-collection filter placed at the outlet of the nebulizer. Such experiments are conducted using a constant breathing pattern provided by a breathing simulator. The breathing patterns recommended by the official compendia are indicative for adult, child, infant and neonate and define values for *V*_T_ (mL), respiratory rate (breaths/min), waveform (sinusoidal), and inhalation/exhalation ratio. Unfortunately, the pharmacopeia do not provide specific requirements for preterm neonates: therefore, these tests may overestimate the dose that reaches an immature patient. The discrepancy between what is required for drug approval and the need to use in vitro settings more representative of the preterm population will be discussed in “Considerations for the development of aerosol therapies for spontaneously-breathing preterm infants” section.

The aerodynamic performance of nebulized aerosol is obtained by studying the APSD: the APSD describes the relative amounts of particles of different sizes within the aerosol. The APSD informs likely deposition site for particles within an aerosol cloud after inhalation. Medical aerosols are made of heterodisperse aerosol particles that usually conform to a log-normal distribution (Fig. 3). They are usually defined by their Mass Median Aerodynamic Diameter (MMAD) and Fine Particle Fraction (FPF). MMAD is the diameter at which half of the aerosolized drug mass lies below the stated diameter, whereas FPF is the fraction of the total drug amount that lies below a specific defined value (usually below 5 µm). The Geometric Standard Deviation (GSD) is also provided as a parameter to describe the variability in terms of particle diameters within the aerosol cloud. Aerosol particles with nearly the same particle size display GSDs < 1.2, whereas GSDs > 1.2 represent heterodisperse aerosols [70]. Thus, the higher the GSD, the broader the particle size distribution.

**Fig. 4:**
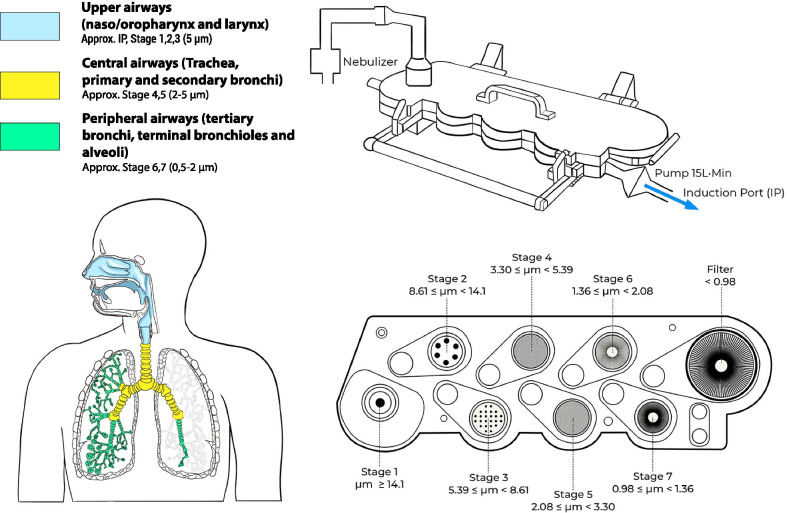
Next Generation Impactor set up and correspondence of the different stages to airway generations

Cascade impactors are the equipment recommended to perform this kind of determination and the Next Generation Impactor (NGI, Copley Scientific) is the standard of choice for this test. The aerosol particles are driven inside the impactor by a constant flow and pass through several stages (cups) with defined cut-offs diameters. This permits the determination of the three main aerosol aerodynamic particle size indicators: MMAD, FPF, and GSD (Fig. 4).

The particle size distribution (PSD) of an aerosol may also be determined by laser-based techniques such as Time of Flight (TOF) and Laser Diffraction [91]. Laser diffraction is a non-aerodynamic optical method for particle sizing that measures droplet volumes by passing the aerosol through a laser beam, resulting in light scattering by the edges of the aerosolized particles. It has to be noted that this method does not determine the actual amount of drug present in the droplets. Nevertheless, for aerosols generated from a solution of a drug, the total volume of the droplets of any given size is proportional to the amount of drug contained within those droplets. This would not be the case for aerosols where the drug is suspended within particles of the aerosol. The usual output variable determined by laser diffraction experiments is the Volume Median Diameter (VMD), defined as the diameter where half of the droplets are larger than this value and half are smaller. VMD is identical to Mass Median Diameter (MMD) if all the particles in the aerosol cloud have the same density as in a nebulized solution.

For the adult population, the reference particle size indicating its ability to reach the lungs has been typically represented by particles with a diameter below 5 µm [50, 70, 92]. For premature infants, however, the reference value remains controversial, although based on previous studies, it can be inferred that such value should be below 2–3 µm. Köhler et al*.* estimated the relative lung deposition of sodium cromoglycate delivered with three different nebulizers (two jet and an ultrasonic nebulizer) by determining the urinary excretion of the compound [39]. The MMDs of the LC Star and LS 290 jet nebulizers were 3.23 µm and 5.04 µm, respectively, while the MMD of the *Project *ultrasonic nebulizer was 3.45 µm. The LC Star achieved the highest lung deposition, which the authors attributed to the higher percentage of aerosol particles with a diameter below 2 µm (20.3%), compared to the LS 290 (6.4%) and the *Project *ultrasonic nebulizer (6.4%). Dubus et al. determined the APSD of ^99m^Tc-DTPA at the outlet of a jet (MistyNeb) and a vibrating-membrane nebulizer (Aerogen Pro) and compared the results with the APSD measured at the outlet of the endotracheal tube (Internal Diameter, ID = 3.0 mm) [77]. The MMAD measured at the outlet of the nebulizer was 4.6 µm for the jet nebulizer; the MMAD of the vibrating-membrane nebulizer was 2.8 µm, if the device was operated in synchronization with the breathing pattern, and 4.8 µm if the device was operated continuously. Interestingly, the MMAD at the outlet of the endotracheal tube was 1.4 µm irrespective of the nebulizer type or operation mode. Similarly, Réminiac et al. determined the MMAD of 99mTc-DTPA aerosols generated by a vibrating-membrane nebulizer (Aerogen Solo) at the outlet of a neonatal-sized nasal cannula at different flows: 2, 4 and 8 L/min [93]. Although the VMD of the aerosol cloud at the outlet of the vibrating-membrane nebulizer was 4.7 µm, the MMADs reported at the outlet of the nasal cannula at flows of 2, 4 and 8 L/min were 1.05 µm, 1.15 µm, and 1.43 µm, respectively. Taken together, these studies suggest that the narrow section of the neonatal ventilation tubes and the conducting airways of premature infants may filter aerosol particles > 2 µm. Therefore, particles compatible with this cut-off diameter would be optimal to achieve a peripheral lung deposition. Moreover, the application of conventional cut off diameters deployed in the characterisation of aerosols for delivery to adults may provide misleading outcomes.

#### Non-invasive respiratory support

**Fig. 5:**
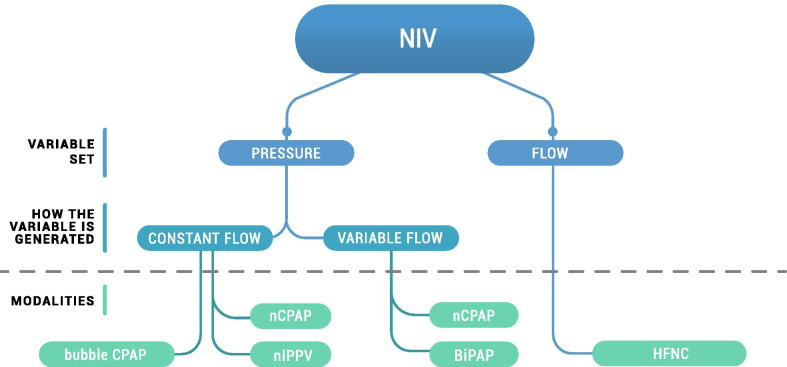
Classification of non-invasive respiratory support—according to the set parameter (pressure vs. flow). Pressure-controlled modalities are classified further based on the pressure generated (constant flow vs. variable flow). The associated ventilatory modalities are reported for each sub-classification. NIV, non-invasive ventilation; CPAP, continuous positive airway pressure; nCPAP, nasal CPAP; nIPPV, nasal intermittent positive pressure ventilation; HFNC, high flow nasal cannula

NIV support modalities are classified into two major categories according to the control parameter set by the operator. The operator sets the targeted delivered pressure in pressure-controlled devices (e.g. CPAP), while the operator sets the flow in flow-controlled modalities (Fig. 5).

Pressure-controlled devices are classified further according to whether pressure is generated by constant or variable flow. The example of pressure-controlled constant flow devices addresses most of the main concepts. Most of comments arising are extendable to all the other devices mentioned in Fig. 5, given that aerosol transport relies on the same hypotheses and mechanisms for each device. The impact of leakage at the interface is not considered for any of the devices described in this section; leakage is discussed in “Nebulizer between the Y-piece and the patient interface” section.

It is worth noting that paediatric ventilators work on a different principle: all the flow passing through the inspiratory limb is directed to the child and therefore the drug is not diluted as occurs with the pure neonatal ventilators. Previous papers describe higher lung deposition for paediatric ventilators in the same configuration (nebulizer along the inspiratory limb) [94, 95]. However, given the important difference in the ventilatory system of paediatric ventilators compared to neonatal ventilators, these results cannot be translated to the preterm population and are beyond the scope of this review.

The following subsections describe the principles and characteristics of the NIV strategies used in preterm infants. This detail will establish the baseline knowledge required to understand how the nebulizer position within the respiratory circuit influences the potential inhaled dose.

##### Pressure-controlled devices with constant flow

###### Standard ventilators

**Fig. 6:**
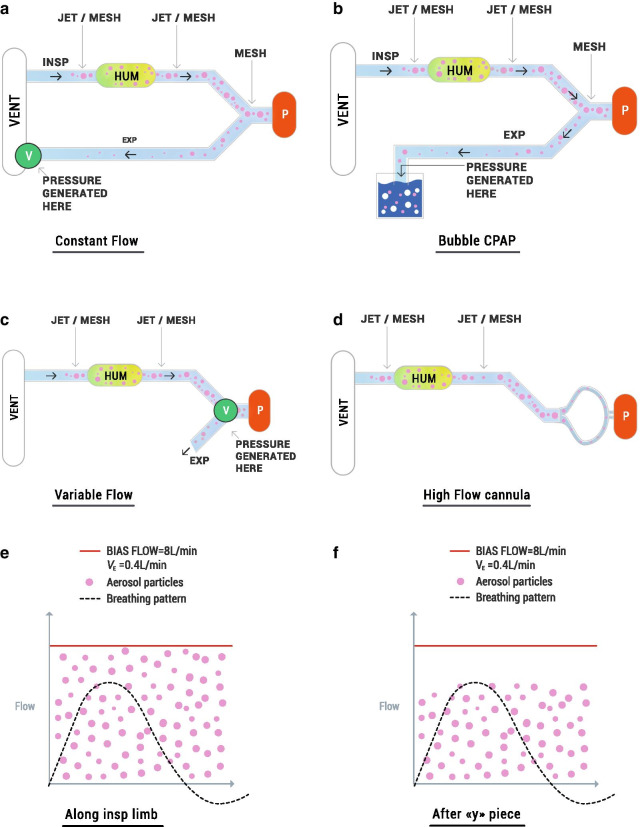
Schematic representation of nebulizer potential position in different NIV systems (**a**–**d**) and the effect on the amount of inhalable of drug (E–F). V = valve, that is the element used to produce the pressure when crossed by the flow, P = patient, HUM = humidifier. The dashed line represents the flow of one representative breath. The red line represents the bias flow for a standard mechanical ventilator in proportion to the breathing flow. Pink dots are aerosol particles produced by the nebulizer and dispersed into the flow. Aerosol particles are distributed directly into the bias flow when the nebulizer is placed along the inspiratory limb (**e**). In contrast, aerosol particles are not removed by the bias flow and are moved only by the breathing flow of the baby when the nebulizer is placed between the patient Y-piece and the airway opening (**f**). In principle, only the particles that can be inhaled are particles produced by the nebulizer and suspended in the airflow during inspiration. This concept is represented graphically by particles enclosed in inspiration: the concentration of the particles is greater when the nebulizer is placed between the Y piece and the airway opening, compared to when the nebulizer is positioned within the inspiratory limb

Standard ventilators (Fig. 6a) are two-limb machines (e.g. Fabian HFO, Acutronic Medical Systems Ag, Hirzel, Switzerland; Dräger Babylog VN500, Dräger Medical System Inc., Andover, MA) that provide mechanical ventilation as well as NIV (e.g. nCPAP and nasal Intermittent Positive Pressure Ventilation, nIPPV). Once the operator sets a pressure, the machine consequently generates a constant flow of air at the desired pressure via a valve, commonly called a positive end expiratory pressure (PEEP) valve. The machine-generated air flow, called bias flow, commonly ranges from 6 to 10 L/min. Uninspired air containing aerosolised drug is directed to the expiratory (PEEP) valve via the expiratory limb in constant flow systems, representing wasted or non-therapeutic drug delivery.

###### Bubble CPAP

Bubble CPAP machines (Fig. 6b) are easy-to-use and inexpensive devices that provide a highly variable instantaneous pressure around a constant mean pressure support [96]. The pressure is generated by coupling a breathing circuit with a constant flow and the immersion of the expiratory limb in a water reservoir. Mean pressure is modulated by changing the depth of the expiratory limb in the water column. In this case, the exhaled drug will be carried into the water reservoir.

###### NIPPV

nIPPV is a form of non-invasive ventilatory assistance using a nasal interface to deliver intermittent positive pressure ventilation to provide respiratory support [97]. In order to provide two levels of pressure, the ventilator acts on the PEEP valve while the bias flow is kept constant.

###### Pressure-controlled devices with variable flow

Variable-flow devices (Fig. 6c) generate the targeted pressure due to a specific valve-interface piece. The first valve used was the Benveniste, then the Infant flow valve has become more popular [96]. A pressure proportional to the flow is generated as the flow passes the valve. Therefore, the pressure is modified by changing the flow (i.e. if 8 L/min flow is needed to produce 5 cmH_2_O, then a 11 L/min flow will be required to produce 8 cmH_2_O). Unlike the constant flow generators, flow and CPAP levels are interconnected in variable flow generators. Thus, the flow must be adapted to ensure that the patient receives the appropriate CPAP pressure. Another important difference of variable flow systems compared with ventilator-controlled flow devices is that the expiratory limb is not connected to the device releasing the expiratory flow. Hence, the aerosolised drug is exhaled directly into the environment when variable flow systems are used.

###### Bilevel CPAP (BiPAP)

Bilevel CPAP (BiPAP) provides two levels of positive airway pressure during the respiratory cycle of the patient with a frequency and a duration determined by the physician [98]. This dual pressure level is achieved by changing the flow delivered to the baby.

###### Flow-controlled devices

The flow-controlled device category consists mainly of devices designed for heated humidified high flow nasal cannula (HFNC) (Fig. 6d). The operator sets the flow to be delivered to the baby instead of the level of pressure because HFNC relies on different working principle as compared to nCPAP [99]. Starting flow levels are commonly from 4 to 6 L/min but flows can be increased up to 10 L/min [100–102]. The pressure delivered to the infant is a function of flow, infant size, degree of mouth closure, and the proportion of the nasal orifice occupied by the nasal cannula. The non-inspired surfactant will leak directly into the environment around the prongs, or out through the mouth.

##### Position of the nebulizer in the respiratory circuit

###### Nebulizer in the Inspiratory Limb

Both jet and vibrating-membrane nebulizers can be placed in the inspiratory limb of all types of ventilators (constant flow, bubble CPAP, variable flow and flow-controlled, Fig. 6a–d). This position significantly decreases the amount of nebulized drug inhaled by the patient, since the amount of drug inhaled is influenced primarily by the bias flow and the minute ventilation (*V*_E_), and to a lesser extent by the breathing pattern [103]. The inhaled dose is estimated as the percentage of the flow inhaled by the baby against the total flow in which the aerosol is diluted, corresponding to the bias flow when the nebulizer is placed within the inspiratory limb (Fig. 6e). Therefore, a higher amount of drug can be inhaled at lower bias flow; similarly, higher amounts of drug may be inhaled at higher minute ventilation assuming that the aerosol particles are distributed homogeneously into the airflow, and that tidal volume effectively clears upper airway dead space (Table 2). Importantly, the percentages of inhaled drug refer to the amount that can be inhaled not the amount that reaches the lungs.

**Table 2:** Estimated maximum inhaled dose for constant and variable flow devices

Patient weight (kg)	Minute ventilation (L/min)	Constant flow			Variable flow	
		Bias flow (L/min)	Inhaled dose (%)		Variable flow (L/min)/CPAP level (cmH_2_O)	Inhaled dose (%)
			Standard ventilator	Bubble CPAP		
0.7	0.3	6	5.0	–	9/5	3.3
		8	3.8	3.8	11/8	2.7
1.0	0.4	6	6.7	–	9/5	4.4
		8	5.0	5.0	11/8	3.6
2	0.65	6	10.8	–	9/5	7.2
		8	8.0	8.0	11/8	5.9

Estimated maximum inhaled dose with the nebulizer in the inspiratory limb, in the ideal case of no air-leak, considering constant flow and variable flow devices. Constant flow: these values do not vary if different CPAP pressures are applied since bias flow level is independent from the pressure that is set for the patient. In the case of bubble CPAP only, a constant flow of 8 L/min is considered (most commonly used and recommended by manufacturers). Variable flow: the authors selected two common CPAP levels and the flow necessary to generate it according to what is reported by the manufacturer (Infant Flow, Vyaire Medical, Mettawa, IL)

A different case is represented by flow-controlled devices (such as HFNC) which have two important drawbacks that significantly limit the possibility of being coupled with a nebulizer. Namely, (1) the leakage at the level of the nose required for this type of respiratory support is so high that the risk of drug loss to the environment is much higher than in other CPAP modalities; (2) the patient interface consists of long cannulas that increase the amount of drug loss due to aerosol condensation on the walls of the tubing, and; (3) the patient inhales only a small percentage of the high flow passing the nebulizer.

##### Nebulizer between the Y-piece and the patient interface

Only vibrating-membrane nebulizers can be put between the Y piece and the patient interface (Fig. 6a, b) and they can only be coupled to constant flow and bubble CPAP ventilators. In principle, vibrating-membrane nebulizers could also be coupled with variable flow systems. However, since such ventilatory systems work with specific patient interfaces the authors are unaware of any existing commercially-available connector to allow this configuration.

The bias flow does not affect the proportion of drug inhaled when positioning the mesh nebulizer between the Y piece and the patient interface, whereas the proportion inhaled can still be affected by minute volume. The theoretical dose inhaled when the nebulizer is placed between the Y-piece and the patient interface should be the total amount of drug nebulized during inhalation, since the only flow passing through the nebulizer is the breathing flow of the baby (Fig. 6f). Therefore, considering an inspiratory time / total inspiratory and expiratory time ratio (Ti/Ttot) of approximately 1:3 [64], this would represent a maximum inhaled dose of 33%. However, this rough estimation is based on two hidden assumptions: (1) there are no air leaks at the patient interface; (2) the inspiratory volume is large enough to replace the gas inside the nebulizer with each breath. Nevertheless, these assumptions are not necessarily true in case of NIV in neonates since leakages are common and, generally, the inner volume of the nebulizer often approaches the tidal volume of the infant. Tarantini et al*.* have recently developed a compartment-based mathematical model that allows estimating the impact of air leaks and nebulizer volume on the lung dose during NIV in premature neonates [104]. For instance, air leaks reduce the amount of drug delivered to the patient, which is released in the environment, whereas the internal volume of the nebulizer may act as a drug reservoir if the tidal volume of the infant is lower than the volume of the nebulizer. The mathematical model showed that in the case of a reduced nebulizer washout (i.e. tidal volume < nebulizer volume) there is an increase of the aerosol concentration within the nebulizer that makes the overall aerosol transport from the nebulizer to the patient system less sensitive to changes in duty cycle but much more sensitive to air leaks. The mathematical model revealed the detrimental effects of air leaks on lung deposition; the authors postulated that the 14% surfactant lung dose estimated by Bianco et al*.* in a bench study conducted using a tightly-sealed neonatal NIV circuit (no air leaks), an eFlow Neos nebulizer, and the breathing pattern of a premature infant [105] would be dramatically reduced to as low as 5.6% in a clinical scenario if the air leaks at the patient interface were considered.

##### Non-invasive ventilation interfaces

Interfaces represent the connections between the baby and the machine providing respiratory support. The most common interfaces used in non-invasive respiratory support are short bi-nasal prongs, nasal masks, and nasal cannula. Also, long nasopharyngeal tubes and a single nasal canula were used previously. Short binasal prongs are superior in terms of maintaining pressure and minimizing inspiratory resistance compared to a single nasal canula and to long nasopharyngeal tubes [106]. Interface type could impact the dose inhaled through three different mechanisms: (1) air leaks; (2) turbulence, and; (3) accumulation of aerosol. Interfaces for NIV do not provide a tight seal with the nose leading to substantial air leaks unless additional measures are used such as colloid protection for the nares that reduces the area for air leaks [107]. Assuming that aerosol particles are homogeneously suspended into the airflow in the airway of the baby, the leaked flow results in an immediate reduction of the inhaled dose. Standard clinical practice requires leakage minimization, but the main variable controlled is the pressure delivered to the baby and not the flow (as flow is often not measured). Ventilators are only capable of providing reliable CPAP with air leaks up to 2 L/min, suggesting larger leakages may be present in clinical settings [108]. In contrast, leakage is needed for the proper working of high flow systems, leading to certain loss of aerosolized material [109]. However, as previously discussed, high flow systems are unsuitable for delivery of an aerosolized drug [109]. Long nasopharyngeal tubes may reduce the leakage compared to the more commonly used short nasal prongs, but they present other well-known drawbacks that limit their usage such as increased resistance and increased nasal secretions [110], which increase the work of breathing.

Turbulence may also impact aerosol delivery: the likelihood of aerosol deposition into the interface is increased when air flow encounters narrowing points or directional change due to increased turbulence. For instance, in case of the infant flow valve, there are very thin channels in which the flow is strongly accelerated, this may promote impaction of the aerosol on the valve channels and, in turn, it may reduce the potential amount of inhaled dose. Additionally, large volumes in the interface foster the accumulation of aerosol particles that increase the density of the particles in the air inhaled by the baby. As a consequence, the inhaled dose may increase because of increased particle density. A good example of this is reported by Minocchieri et al*.* where a resuscitation mask was used to deliver an aerosol exploiting the accumulation phenomenon [73]. Nevertheless, the evident drawback is the increase of dead space that should be handled carefully to avoid detrimental effects on carbon dioxide exchange. To address this, Minocchieri et al*.* drilled a small hole in the mask to reduce the risk of carbon dioxide accumulation resulting in a small associated loss of surfactant aerosols into the environment. However, a real benefit of this procedure has not been established and no specific masks with this feature have been designed and marketed.

Finally, notably a specific interface currently under investigation has been developed ad hoc for supporting aerosol delivery. The ventilator circuit/patient interface connector (Afectair®; Discovery Laboratories, Inc.) described by Mazela et al*.* in 2014 consists of a modified Y piece that includes a preferred channel directing the therapeutic aerosol from the aerosol generator to the baby to prevent the removal effect of the ventilator’s bias flow, as described in most of the examples above [111]. Additionally, this connector delivers the aerosol to the patient under lower and more laminar flow conditions reducing the impact of turbulence. In an in vitro study, Mazela et al*.* compared this connector with a standard interface (T connector) for albuterol sulfate delivery with a jet nebulizer under neonatal ventilation conditions. They reported a 11.3% delivery of the nominal dose compared to 1.2% delivery using the T connector.

#### Aerosol synchronization

Synchronized nasal intermittent positive pressure ventilation (sNIPPV) is an effective modality in aligning flow with the infant inspiratory phase [112]. The most commonly cited methods of synchronization are the Graseby capsule (GC) and neurally-adjusted ventilator assist (NAVA) [113]. The GC consists of a small polythene foam-filled disk, fixed to the anterior abdominal wall below the xiphisternum, whereas NAVA relies on a diaphragmatic electromyogram [113]. Reduced delay between the neural signal and the flow activation is a key advantage of NAVA.

Other technologies for sNIPPV based on use of a hot wire anemometer flow sensor are available for some countries, albeit less common [113]. The flow sensor is designed to be resistant to high nasal leakage and able to detect the inspiratory trigger. Nevertheless, flowmeters are unsuitable in conjunction with aerosol delivery as their function is impacted negatively by the aerosol particles that deposit on them.

Theoretically, synchronizing the production of an aerosolized drug with inhalation could significantly reduce the amount of drug lost during exhalation and consequently improve the dose delivered to the lungs. Nevertheless, to date, synchronization is not possible when the nebulizer is positioned within the inspiratory limb, as the transit time delay for the aerosolized particles to be driven from point of aerosol generation to the baby is too long to achieve effective synchronization. For instance, placement of the nebulizer proximal to the humidifier, would result in a delay of 2.5 s for the aerosol to exit the humidifier [114] with a standard ventilator flow of 8 L/min. As this delay far exceeds the duration of a breath, aerosol delivery synchronized to inhalation is not possible.

Even if we consider the most favourable case in which the nebulizer is placed after the humidifier, the volume of the tubing is still approximately 120 mL [114]. Transit time along this tubing takes approximately one second at a bias flow of 8 L/min. This delay is still significant in comparison to the inspiratory time of preterm infants and highlights the impracticality of achieving synchronized generation and delivery of aerosol restricted to the inspiratory phase.

The case in which the nebulizer is placed after the Y piece makes synchronization theoretically feasible but there are additional technical challenges related to appropriately sensing the breath onset and the immediate activation of the nebulizer. Good results in terms of lung deposition obtained with synchronization were evident when an atomizing catheter was used in a preclinical setting [81]. Drug deposition of approximatively 30% was reported [81]; however, the administration was done intracorporeally and close to the vocal cords representing the best scenario for synchronization, albeit it is recognised that the approach is slightly more invasive.

#### Non-invasive ventilation circuit humidity and temperature

NIV support to premature infants is delivered as humidified air pre-warmed to physiological temperature in order to protect the airway mucosa from dehydration and maintain its homeostasis. Nevertheless, the humidity of the NIV circuit may increase the size of aerosol particles [115, 116], especially in the case of hygroscopic formulations, and may promote the coalescence of aerosol particles into larger aggregates [88]. These phenomena may, in turn, increase the chance of inertial impaction in the NIV circuit, nasal passages, and upper airways, thereby reducing the effective lung dose. Bianco et al*.* reported a slight but significant increase of the MMD of surfactant aerosols generated with a customized eFlow Neos nebulizer from 2.6 μm at 30% relative humidity, to 3.0 μm at 90% relative humidity, which reduced the FPF from 97.2% to 93.7% [105]. Martin and Finlay generated salbutamol aerosols into a holding chamber with a pMDI and determined the APSD at the outlet of a tube at 15 cm distance from the chamber at both 10% and 100% relative humidity conditions (T = 37 °C in both cases) [115]. At 10% humidity conditions, the fraction of salbutamol deposited in the holding chamber was 38.9% and the MMAD of the aerosol was 1.97 μm (GSD = 1.38); however, adding humidity to the air-flow until saturation significantly increased the fraction of salbutamol deposited in the holding chamber to 50% of the nominal dose and nearly doubled the MMAD to 3.75 μm (GSD = 1.23) [115]. Therefore, aerosol delivery to premature neonates through NIV circuits at low relative humidity conditions could theoretically improve the lung dose by keeping the aerosol particle size low for optimal nasal delivery. Nevertheless, although the need for humidification during NIV remains controversial in adult patients [117], infants should receive conditioned air both in terms of humidity and temperature [9]. To circumvent this limitation, Longest et al*.* proposed a delivery method termed “Enhanced Condensational Growth” (ECG) aimed at increasing the aerosol delivery efficiency through nasal interfaces during NIV [118]. The method consists of delivering submicron aerosol particles and saturated air at 39 °C to the left and right nostrils, respectively. This way, submicron aerosol particles travel through one channel of the nasal cannula, which is fed with dry air, and humidified air is delivered through the other channel. Condensational growth of the aerosol particles begins at the nasopharynx when both dry and humidified air streams mix, increasing the particle size from submicron diameter to approximately 2 μm at the level of the tracheal. This increase in diameter improves the lung retention of the particles. This method reduces extrathoracic aerosol deposition in an adult replica of the upper airways in in vitro and in silico studies and is a potentially interesting approach for aerosol delivery to premature neonates [78, 118]. In this regard, a recent study by Kamga Gninzeko et al*.* used this approach to treat surfactant-deficient rats with a novel formulation of spray-dried pulmonary surfactant composed of *Beractant* (Survanta®), mannitol, and leucine [119]. The spray-dried surfactant formulation was reported to be homodisperse (comprised of particles with highly uniform size) with a particle size diameter of 1.0 ± 0.04 μm. The formulation included mannitol as a hygroscopic excipient, which absorbs water as particles travel through the airways turning the particles in liquid droplets of a higher mass and diameter. Interestingly, the spry-dried surfactant, delivered by a novel dry powder inhaler, achieved a uniform pulmonary distribution and significantly improved lung compliance compared to the group of animals treated with the excipient-free liquid *Beractant* [119]. The in vivo study by Kamga Gninzeko and colleagues thus highlights the potential of this technique for nasal aerosol delivery.

#### Miscellaneous

Several other extrinsic factors may influence pulmonary aerosol deposition in premature infants. For instance, viscous formulations such as exogenous surfactant preparations [120] and liposome-based formulations may affect the performance of nebulizers [121], which in turn requires the selection of appropriate devices. Linner et al*.* reported the need to dilute *Poractant alfa* 1:1 with saline to achieve the optimal performance of an investigational eFlow vibrating-membrane nebulizer [122]; whereas Finer et al*.*, in a clinical study of surfactant nebulization, found marked variability in terms of nebulizer output between the single AeroNeb Pro vibrating-membrane nebulizer units when delivering *Lucinactant* at a concentration of 20 mg/mL [38].

The use of heliox (a gas mixture containing 20% oxygen and 80% helium) as driving gas reduces airway resistance and improves ventilation in mechanically-ventilated patients with airway obstruction [123]. Heliox also increases the effectiveness of nCPAP in premature neonates with RDS, reducing the need for mechanical ventilation [124]. Gas mixtures of helium and oxygen have lower density than equivalent air-oxygen mixtures and may achieve more laminar flow regions in larger airways favouring peripheral lung deposition. In a scintigraphy study, Piva et al*.* compared the distribution of inhaled 99Tc-DTPA in 20 children in the age range 5–15 years-old and reported that heliox led to a better lung deposition of ^99^Tc -DTPA-labeled particles compared to oxygen [125]. The differences in lung deposition were even more pronounced in those patients with severe lower airway obstruction. Nevertheless, the use of heliox has not been evaluated in the context of aerosol delivery to premature infants to date.

Premature infants managed with NIV can be positioned both supine and prone [126]. Repositioning during the course of ventilation may improve ventilation homogeneity and oxygenation [127–129]. However, position changes alter the upper respiratory tract angles and may therefore influence the fate of a nebulized drug. A recent scintigraphy study by Cunha-Goncalves et al*.* in newborn piglets ventilated with nCPAP investigated the impact of body positioning on the lung distribution of nebulized surfactant [130]. In this study, all animals received 200 mg/kg of nebulized, radiolabelled surfactant using a customized eFlow Neos vibrating-membrane nebulizer placed between the Y piece and the nCPAP interface (customized nasal prongs). Four groups of animals (n = 6) received nebulized surfactant in different body positions: prone, supine, lateral decubitus with the right side up, and lateral decubitus with the left side up. The authors found the highest and most reproducible lung deposition (32.4 ± 7.7%) in the group of animals positioned in the prone position (21.0 ± 8.6 in the right lung and 11.3 ± 5.7% in the left lung, mean ± SD). Mean lung deposition in the supine position was half (15.2%; 10.7 ± 11.4% in the right lung and 4.5 ± 2.4% in the left lung) of that achieved in animals positioned in the prone position. The mean lung deposition in the animals positioned in lateral decubitus was 18.7% and 13% for animals with the right side up and left side up, respectively; notably, in both groups of animals positioned in lateral decubitus, more than 80% of the deposited surfactant was detected in the dependent lung, which suggests that surfactant deposition is influenced by gravity.

Another element to be considered when developing a drug for pulmonary administration in premature infants is the constrains related to the formulation definition. Formulations for nebulization usually require excipients. For instance, solution formulations may require excipients to improve chemical stability and prevent degradation of the active ingredients while suspensions may need excipients to optimize physical stability, avoid quick sedimentation or creaming, control flocculation, and prevent the sticking to the primary packaging. Excipients could also be introduced to modulate the aerosol particle size distribution in order to obtain the desired deposition in the respiratory tract or change the drug adsorption and bioavailability. The incorporation of excipients into neonatal medications could be very critical and should be carefully monitored as many excipients considered to be pharmacologically inert in adults may be toxic to neonates [131].

## Clinical studies with nebulized drugs during NIV

The first report of an attempt to nebulize a drug as a treatment for neonates with nRDS dates back to 1964: Robillard and colleagues nebulized a synthetic mixture of lipids using an aerosol generator placed in the incubator [132]. The nebulization period varied from 15 min up to four hours and the authors reported that the respiratory distress was alleviated in 8 out of 11 treated infants [132]. Since then, aerosol and ventilatory technology have advanced considerably, improving the management of respiratory conditions in the neonatal population. Administration of aerosolized drugs in premature infants was initially targeted to those on mechanical ventilation. However, as NIV has become an increasingly standard respiratory support modality for all spontaneously breathing premature infants, so too the focus for aerosolization has shifted to its application during NIV. During NIV, drug delivery may be achieved while disconnecting the patient from the respiratory support for a short period and using pMDIs or jet nebulizers with a facial mask as the delivery interface. However, disconnecting a premature infant from respiratory support for drug delivery may not be the most optimal approach.

Here, we focus on studies that investigated drug nebulization using aerosol generator coupled with non-invasive respiratory support. All such trials published to date were performed with surfactant (Table 3). Treatment efficacy is not discussed in detail, as study sample sizes are too small, and used a range of surfactant preparations that have different efficacy profiles [79, 133]. Hence, the focus of the discussion highlights how the selected NIV modality, nebulizer type, and its position in the respiratory circuit potentially influenced the amount of inhaled drug.

**Table 3:** Summary of clinical attempts to nebulise surfactant during non-invasive ventilation

Study (author—year)	Patients (GA in weeks and BW in Kg)	Nebulizer type	Ventilation	Nebulizer position	Interface	Drug	Particle size
Jorch et al*.* 1997	31 (28–35) BW N.A:	Jet	Bubble CPAP	Y piece	Nasopharyngeal tube	Animal derived surfactant Bovactant	< 4 µm (98% of the particles)
Arroe et al*.* 1998	23–36 BW N.A	Jet	nCPAP	Inspiratory limb	Unknown	Animal derived surfactant—colfosceril palmitate	N.A
Berggren et al*.* 2000	27–34 1.62 (1.01–2.37)	Jet	Infant flow	inspiratory limb	Prongs	Animal derived surfactant –	< 2 µm
Finer et al	28–32 1.50 (1.00–2.30)	Vibrating-membrane	Several NIV types	Y piece	Prongs	Synthetic surfactant—KL4	N.A
Guardia et al*.* 2018	29–34 BW unknown	Capillary Aerosol Generator (CAG)	Bubble CPAP	Y piece	Affectair®	Synthetic surfactant—KL4	N.A
Sood et al*.* 2019	24–36 0.79–2.25	Jet	nCPAP/nIPPV unspecified driver	Inspiratory Limb	N.A	Animal derived surfactant—Beractant	N.A
Minocchieri et al*.* 2019	29–33 1.56 (mean)	Vibrating-membrane	Bubble CPAP	Y piece	facial mask (with hole)	Animal derived surfactant—Poractant alfa	2.6 µm (MMD)
Cummings et al*.* 2020	23–41 1.96 (0.59–4.80)	Jet	Several NIV types	Inside the mouth	Pacifier adapter	Animal derived surfactant—Calfactant	N.A

nCPAP, nasal continuous positive airway pressure; N.A., not available; NIV, non-invasive ventilation; nIPPV, nasal intermittent positive pressure ventilation; MMD, mass median diameter

The first clinical study of a nebulized surfactant coupled with NIV was described by Jorch et al*.* in 20 premature infants (28–35 weeks GA) managed on pharyngeal bubble CPAP in an uncontrolled study [71]. Nebulized surfactant (*Bovactant*, Alveofact®, Lyomark Pharma, Obehaching, Germany) was administered via a jet nebulizer (RO252/ME, Intersurgical, Wokingham, UK) connected directly to a T piece between the inspiratory limb and a nasopharyngeal tube. A rapid improvement in alveolar-arterial gradient (A-a DO_2_) was observed and only six out of 20 patients required intubation. However, increased secretions were common adverse events. Notably, jet nebulizers produce fast-moving particles that need to be slowed down to limit impaction. The position of the nebulizer so close to the airway was probably chosen to reduce drug losses, but likely resulted in a considerable amount of drug impacting in the nasopharyngeal tube. Impacted surfactant would reach the pharynx in liquid form rather than as an aerosol, promoting drug accumulation in the upper airways and not in the lung.

Arroe et al*.* reported the treatment of 22 infants (22–36 weeks GA) with a Sidestream 45 jet nebulizer placed in the inspiratory limb of the circuit [37]. No therapeutic effect of the treatment was reported. Absence of surfactant proteins in the synthetic surfactant used (*Cosfoceril palmitate*, Exosurf®) and minimal inhaled drug dose due to the nebulizer type and position likely contributed to the lack of clinical effect.

Berggren published data from a small non-randomised trial in 34 infants (27–34 weeks GA) comparing CPAP alone to CPAP plus nebulized surfactant. Unlike earlier studies, the applied technology was described in detail [72]. A jet nebulizer (Aiolos, Karlstad, Sweden) was positioned in the inspiratory limb of a variable flow ventilator (Infant Flow, Dansjö Medical AB, Bromma, Sweden) with a flow of 7 L/min and a nebulization rate of 0.2 mL/min. Treatment took approximately 3 h to nebulize a total amount of 34 mL of study medication (*Poractant alfa*, Curosurf®, 480 mg diluted with saline to a 20 mg/mL concentration). The authors verified that drug maintained its surface tension properties on a Wilhelmy balance before study initiation. Although surfactant nebulization was safe, the study results were negative. And indeed, the authors commented that an important factor they could not measure was lung deposition in the patients. As a surrogate, they measured lung deposition following nebulization in a rat model. The amount of exogenous phospholipids in post-mortem lung lavages after surfactant aerosolization accounted only for 0.5% of the nominal surfactant dose. However, the authors reported a significant improvement in arterial oxygenation after nebulization, although aerosolized surfactant remained significantly inferior to bolus endotracheal surfactant administration [134]. Nevertheless, the animal study was conducted in mechanically-ventilated rats and therefore air leaks could be controlled compared to the clinical study: these factors most probably accounted for an even lower lung deposition in the clinical study.

Nebulization using a vibrating-membrane nebulizer was first reported by Finer et al*.* [135]. The nebulizer was placed at the Y piece using a special connector to avoid bias flow associated loss of surfactant directly into the expiratory limb when connected to either bubble CPAP or synchronized nasal intermittent ventilation. The trial aimed to study two different surfactant regimens given prophylactically in 20 preterm infants stratified according to their gestational age (28–29 and 30–32 weeks GA) treated with 20 mg/mL of synthetic surfactant (*Lucinactant*) over a 3-h period, with up to 3 surfactant retreatments allowed. The produced aerosol had a low MMD (1.9 ± 0.3 µm) with an aerosol output rate up to a maximum of 0.4 mg/min. The authors claimed that these conditions would allow surfactant delivery to an infant of up to a maximum total dose of 72 mg; close to the dose provided by endotracheal administration. Study results show a transient beneficial effect, different from what is observed with endotracheal surfactant. However, the real lung dose may have been considerably less than stated due to short neonatal inspiratory times and leakage at the patient interface. Indeed, the low lung delivery may explain the lack of sustained efficacy.

Recently, a phase I safety study reported aerosolization of a surfactant via a jet nebulizer placed in the inspiratory limb of different ventilatory systems (high flow nasal cannula, nCPAP, or nIPPV with unspecified ventilators) [74]. The study results show a good safety profile in infants above 29 weeks GA but has two major limitations: 1) drug nebulization is incompatible with humidified high flow (see “Position of the nebulizer in the respiratory circuit” section), and 2) the inhaled drug dose is very low when the nebulizer is positioned in the inspiratory limb, even without considering air leaks at the patient interface. Therefore, intrapulmonary surfactant deposition was likely negligible for patients in this study.

A further recent trial by Minocchieri et al*.* describes a very different setting consisting of a vibrating-membrane nebulizer positioned in a bubble CPAP system after the Y piece and connected to the patient via a face mask [73]. Premature infants (29–32 weeks GA) were randomised to CPAP only or CPAP plus surfactant (*Poractant alfa*). Importantly, the treating clinical team and the parents were unaware of the treatment received. Relative risk for intubation in the first 72 h was reduced in the surfactant group; further analysis showed the reduction in intubation was restricted to 32–33 weeks GA infants only. The study is limited by its small size. There was also a higher than anticipated intubation frequency in the control group and the more immature surfactant group: a frequent unit practice at the time of study conduct (2010–2012) was to intubate early for bolus surfactant administration if respiratory distress persisted even in the absence of hypercarbia or requirement for substantial supplemental oxygen. Hence this study remains inconclusive.

More recently, Cummings et al*.* have reported the results from the trial comparing the oral aerosolization of *Calfactant* with the standard care in infants with RDS [86]. This study enrolled 457 infants (23–41 weeks GA), representing the largest trial on surfactant aerosolization conducted so far. The authors reported a significant decrease in the proportion of newborns intubated for liquid surfactant instillation in the intervention group. Although the results appear to be encouraging, the trial has a few shortcomings that give rise to diverse interpretations. Glaser and Wright identified some important potential sources of bias in the study design, including the lack of a clear criteria for liquid bolus surfactant therapy and the absence of a strict definition of failure of the intervention with aerosolized surfactant [136]. They also raised a concern on the limited inclusion of infants < 28 weeks’ gestation.

The potential systemic exposure of the infant to a drug is an important factor when developing an aerosol dosage form for premature infants: the fate of the remaining aerosol needs to be considered as only a small fraction of the aerosol gets through the vocal chords. As such, the position of the nebulizer in the respiratory circuit and the type of ventilatory setting play an important role. A substantial portion of the drug is carried out by the bias flow in the expiratory limb when the nebulizer is placed either in the inspiratory limb or (to a lesser degree) after the Y piece. However, even placement of the nebulizer between the Y piece and nasal prongs does not guarantee drug delivery to the lung. Animal studies reported by Nord et al*.* using *Poractant alfa* show that 19% of the drug delivered to the upper airway is deposited in the nose and pharynx, and an additional 12% is traced to the stomach [137]. The distribution of drug to the pharynx and stomach may be higher when drug is delivered to the oropharynx using atomizing catheters, with the possibility of either local effects or systemic absorption. Therefore, the pharmacokinetics of drug absorption into the bloodstream should be studied carefully to ensure procedural safety: such studies are especially important when investigating potential side effects of the procedure, in particular when investigating drugs such as corticosteroids for which significant adverse side effects with systemic exposure are widely acknowledged [138, 139].

While the studies reported above provide important insights and some encouraging data about surfactant nebulization, intrinsic and extrinsic factors are likely to have impacted significantly on lung deposition and therefore on the pharmacological, and in turn on the clinical, effect. Overall, all studies showed a good safety profile. Three studies described some therapeutic response [71, 73, 135], and unfortunately there was only one randomized controlled trial [73]. Statistically significant benefit was only observed in the higher GA groups: in all cases the nebulizer was placed just before the patient interface. We suspect that the physiological effect observed by Sood et al*.* is unlikely attributable to the drug, since surfactant was delivered by a jet nebulizer placed in the inspiratory limb, and which therefore led to a series of extrinsic factors limiting lung delivery as presented above [74].

## Considerations for the development of aerosol therapies for spontaneously-breathing preterm infants

### Limitations of the regulatory guidelines

The US Pharmacopeia and the European Pharmacopoeia offer guidelines on the development of aerosol medicines [89, 90]. These documents provide guidance on how to evaluate the quality of different drug product and device batches. However, these methods do not take full account of the specific patient population characteristics or the clinical environment in which the aerosolization is performed: both factors can have a significant impact on the aerosol characteristics and ultimately on the dose delivered to the lungs. In particular, we want to highlight the mismatch between compendial requirements and the prenatal population with regards to breathing pattern, patient interface, environment temperature, nebulizer position, the combination of a nebulizer within the respiratory circuit, the way APSD should be studied, and the connection between the nebulizer and the in vitro equipment (Table 5).

The breathing pattern of the smallest patient population proposed by the official compendia (the “Neonate”) is not representative of the preterm population. Similarly, compendia prescribe the use of a mouthpiece as the patient interface whereas preterm neonates are preferential nose breathers and the typical interfaces are nasal prongs and nasal masks.

Preterm neonates, especially the smallest ones, are typically kept in an incubator with substantially different environmental conditions (e.g., incubator temperature, high air humidity, and warm air temperature) from those proposed in the compendial tests. Existing guidelines do not consider the connection of a nebulizer with a ventilator system. Consequently, evaluations do not consider the effect of the ventilation parameters (ventilator bias flow, air pressure, air humidity, air temperature) on the aerosol generation and transportation. The mismatch between guidelines prescriptions and real life becomes striking when considering the aerodynamic assessment of nebulized aerosol (NGI test): the NGI test requires a constant flow of 15 L/min inside the device and the NGI equipment. It represents the mid-inhalation flow rate achievable by a healthy adult individual with a 500 mL *V*_T_. Finally, the pharmacopoeia prescribes air-tight connections between the nebulizer and the testing equipment (both NGI and breathing simulator), while in clinical practice the leakages usually play an important role in the amount of drug that reaches the lungs. Table 4 summarizes the main differences between the parameters described in the compendial method for the smallest patient size (Neonate) and the proposed parameters for premature neonates.

**Table 4:** Comparison of the parameters required by compendial methods and those representative for the premature population

	Compendial method “Neonate”	Premature population: Parameters proposed for “Premature Neonate”
Breathing pattern	*V*_T_: 25 mL RR: 40 breaths/min I:E ratio: 1:3 Waveform: sinusoidal	*V*_T_: 6–8 mL RR: 60–90 breaths/min I:E ratio: 2:3 Waveform: variable/sinusoidal
Nebulizer temperature (temperature at which the nebulizer is operated)	23 ± 3.0 °C (controlled lab temperature)	Incubator temperature typically up to 37 °C
Air temperature	23 ± 3.0 °C (controlled lab temperature)	Warm air, up to 37 °C in the incubator and NIV circuit
Air humidity	Ambient air humidity	Humid air, typically 95–99% of relative humidity
Airflow inside the nebulizer	15 L/min	2–8 L/min
Connection of the nebulizer to a ventilatory driver	No	Yes
Patient interface	Mouthpiece	Prongs, mask, or other neonatal interfaces

The parameters are proposed by the authors as an example of an average patient and are not representative of the variability that can be observed in the heterogeneous premature infant population. *V*_T_, tidal volume; RR, respiratory rate; I:E ratio, inspiratory-expiratory ratio

### In vivo models

The ideal in vivo model to mimic aerosol delivery to spontaneously-breathing preterm infants would include the features of the immature respiratory system. These features include surfactant deficiency, immature lung structure, low lung compliance, high tissue resistance, and periodic breathing. The model should have airways that are similar in size and calibre to those of premature infants and studies should be conducted using NIV (ideally with devices used at the neonatal intensive care unit, NICU), and use the same nebulizers intended to be used in humans. The model and preclinical facility would allow testing of the efficacy as well as the lung deposition and the pulmonary distribution of aerosolized drugs. Such an idealized animal model is unfortunately not available and therefore several animal models with distinctive features must be used to address the different aspects of the feasibility of the therapy, the pharmacology, and the lung deposition and distribution of a given aerosolized drug.

The most commonly employed animal models displaying premature lungs are lambs and rabbits delivered before term by caesarean-section. The mechanically-ventilated premature lamb model played a central role in the development of surfactant therapy [140–142] and has been the gold standard model to mimic the initial pathophysiological steps of RDS. It has been used to compare the efficacy of different surfactant types after intratracheal instillation [56, 143–146], to test the efficacy of anti-inflammatory drugs [20, 21, 147, 148], to gain insights onto the initial steps of the lung inflammatory process in the context of RDS [149, 150], and to investigate the lung and cerebral effects of intravenously-delivered drugs [151–153], among others. Interestingly, spontaneously-breathing premature lambs can be supported with NIV [81, 154], monitored with NICU equipment, and allow the integration of nebulizer devices within the NIV circuit [155]. Rahmel et al*.* established a spontaneously-breathing preterm lamb model supported with CPAP delivered by binasal prongs [87]. The authors used this model to investigate the feasibility and the tolerability of a novel Continuous Powder Aerosolization (CPA) system that generated humidified recombinant surfactant protein C surfactant aerosols from a dry powder formulation [88]. Similarly, Hütten et al*.* investigated the efficacy of nebulized *Poractant alfa* surfactant delivered by a customized vibrating-membrane nebulizer to spontaneously-breathing preterm lambs during binasal CPAP [156]. Therefore, the preterm lamb model can be considered as a useful model for late-stage preclinical development of aerosol therapies intended for spontaneously-breathing preterm neonates. Nevertheless, this model also has some limitations: it requires significant resources and shows a marked inter-subject variability in terms of pulmonary status at birth (even at controlled gestational ages), often requiring larger sample sizes that in turn make the model less cost-effective.

Premature rabbit foetuses delivered at 27 days of gestation display a severe surfactant deficiency, require mechanical ventilation for survival, and usually have a very limited life-span [24, 157, 158]. However, if the premature fetuses are delivered at 28 days of gestation, the animals have enough respiratory drive to breathe spontaneously, while they exhibit structural and functional lung immaturity [159]. Moreover, premature rabbits exposed to hyperoxia for a few days develop a BPD-like phenotype [160] and provide a suitable long-term model to test the efficacy of active molecules for the prevention and treatment of BPD [161–164]. Salaets et al*.* recently established such a model demonstrating the feasibility and the safety of performing daily intratracheal injections of saline and surfactant [165]. This model represents a promising tool to evaluate topical pharmacological interventions for the treatment of BPD. Unfortunately, preterm rabbits are very small (birth weight ~ 20–40 g) compared to human premature neonates and, so far, they have not been managed with clinical NIV devices nor have they been used in aerosol studies.

In view of the challenges posed by premature animal models, alternative models, including spontaneously-breathing adult and paediatric models, have been employed in the context of neonatal aerosol delivery. Most of these models were established to assess the efficacy of nebulized surfactant and therefore rely on either depleting or inactivating the endogenous pulmonary surfactant to produce a respiratory distress that could theoretically be reverted by the treatment with nebulized surfactant. For instance, surfactant-depleted adult rabbits weighing 1.5–2.5 kg approximate the weight of premature infants and can be managed with different NIV modalities using a neonatal ventilator [166]. This model was used widely for the efficacy testing of natural and synthetic surfactants delivered using jet nebulizers, vibrating-membrane nebulizers, and investigational aerosol generators [36, 105, 167–169]. Newborn piglets less than 1 week of age and body weights ranging from 1.2–2.0 kg have also been used in aerosol studies in the context of RDS [32, 170]. The small size of the piglets makes them a good model to mimic the small airway diameter of the premature infants. In newborn piglets, respiratory distress can be generated by either instilling HCl intratracheally [171, 172], which inactivates the endogenous surfactant and creates a diffuse lung injury, or by repeated broncho-alveolar lavages that deplete the intrapulmonary surfactant pools [173]. Rey-Santano et al*.* recently described a spontaneously-breathing, surfactant-depleted piglet model supported with NIV in which they nebulized undiluted surfactant (*Poractant alfa*, 80 mg/mL) with an eFlow Neos nebulizer customized to neonatal standards [174]. Interestingly, they expanded the follow up period up to 72 h and in addition to the pulmonary effects, they also investigated the hemodynamic response and the cerebral effects after delivery of nebulized surfactant [174].

A common limitation in most in vivo studies is the lack of data regarding lung deposition and pulmonary distribution of nebulized drugs. Considering that lung deposition correlates with inhaled drug efficacy, reliable data on lung deposition and distribution in relevant preclinical models are among the best predictors of the clinical potential of an aerosol therapy. These investigations, however, often require dedicated experiments (i.e. no other outcome than lung deposition and distribution can be obtained) and might be limited, at least in part, by the restricted access of preclinical research groups to the required imaging facilities. Table 5 describes selected preclinical studies that aimed to determine the lung deposition and distribution of nebulized drugs in relevant neonatal animal models.

**Table 5:** In vivo studies of radiolabelled aerosol deposition and distribution under neonatal ventilation conditions

Study	Model, body weight	Nebulizer type and position	Nebulized substance	Particle size	Ventilation type	Interface	Lung deposition (% of the nominal dose)
Dubus et al*.* 2005	Healthy macaques, 2.5–2.8 kg	a) MistyNeb (Jet) at inspiratory limb b) Aeroneb Pro (VM) (continuous or sync) at Y-piece	^99m^Tc-DTPA	MistyNeb = 4.6 μm Aeroneb Pro (cont.) = 4.8 μm Aeroneb Pro (sync) = 2.8 μm	Mechanical ventilation	ET tube (ID = 3 mm)	Mistyneb = 0.5 (0.4—1.3)* Aeroneb Pro (cont.) = 12.6 (9.6—20.6)* Aeroneb Pro (sync) = 14.0 (12.2 – 23.7)*
Linner et al*.* 2014	Healthy newborn piglets, 1.2–2.2 kg	Investigational eFlow nebulizer (VM) at Y-piece	^99m^TC-labelled *Poractant alfa* diluted 1:1 with saline	MMAD = 2.5–3.0 μm (GSD = 1.6)	nCPAP (4 cmH_2_O) PPV	Mask, prongs, and ET tube (ID = 2.5 or 3 mm)	Mask (nCPAP) = 5 (3 – 13)* Prongs (nCPAP) = 14 (2 – 40)* ET tube (PPV) = 45 (25–56)* *Intratracheal instillation* = 88 (71–96)*
Réminiac et al. 2016	Healthy macaques, 3.2–3.6 kg	a) Cirrus2 (Jet) at inspiratory limb b) Aerogen Solo (VM) at inspiratory limb	^99m^Tc-DTPA	Cirrus2 = 4.0 μm Aerogen Solo = 4.7 μm	HNF (8, 4 and 2 L/min)	Nasal Cannula	Cirrus2, 8 L/min = 0.03 ± 0.03^§^ Aerogen Solo, 8 L/min = 0.09 ± 0.04^§^ Aerogen Solo, 4 L/min = 0.49 ± 0.44^§^ Aerogen Solo, 2 L/min = 0.85 ± 0.57^§^
Gregory et al*.* 2019	Healthy macaques, 4.8–6.8 kg	Aerosurf delivery system (prototype) at Y-piece	^99m^TC-labelled *Lucinactant* (surfactant)	MMAD = 2.91 μm (GSD = 1.81)	nCPAP (5 cmH_2_O)	Prongs with Affectair®	7.52 (3.53 – 14.07)^#^
Nord et al*.* 2020	Healthy newborn piglets, 1.2–2.2 kg	Customized eFlow nebulizer (VM) at Y-piece	^99m^TC-labelled, undiluted *Poractant alfa*	MMD = 3.0 μm GSD = 1.5	nCPAP (3 cmH_2_O) nIPPV (3 + 3 cmH2O)	Prongs	nCPAP = 15.9 ± 11.9^§^ nIPPV = 21.6 ± 10.0^§^
Cunha-Goncalves et al*.* 2020	Healthy newborn piglets, 1.3–2.2 kg (Treated either in supine, lateral decubitus, or prone position)	Customized eFlow nebulizer (VM) at Y-piece	^99m^TC-labelled, undiluted *Poractant alfa*	MMD = 3.0 μm GSD = 1.5	nCPAP (3 cmH_2_O)	Prongs	Prone (right lung) = 21.0 ± 8.6^§^ Prone (left lung) = 10.7 ± 11.4^§^ Supine (right lung) = 10.7 ± 11.4^§^ Supine (left lung) = 4.5 ± 2.4^§^ Right side up (right lung) = 3.4 ± 1.0^§^ Right side up (left lung) = 15.3 ± 13.4^§^ Left side up (right lung) = 11.2 ± 9.8^§^ Left side up (left lung) = 1.8 ± 0.7^§^

VM, vibrating-mesh; ^99m^Tc-DTPA, Technetium-99 m diethylenetriaminepentaacetic acid; MMAD, mass median aerodynamic diameter; GSD, geometric standard deviation; nCPAP, nasal continuous positive airway pressure; HNF, humidified nasal flow; MMD, mass median diameter; nIPPV, nasal intermittent positive pressure ventilation; ET tube, endotracheal tube; ID, internal diameter. *Median (range); ^§^ Mean ± SD; ^#^ Mean (range)

**Fig. 7:**
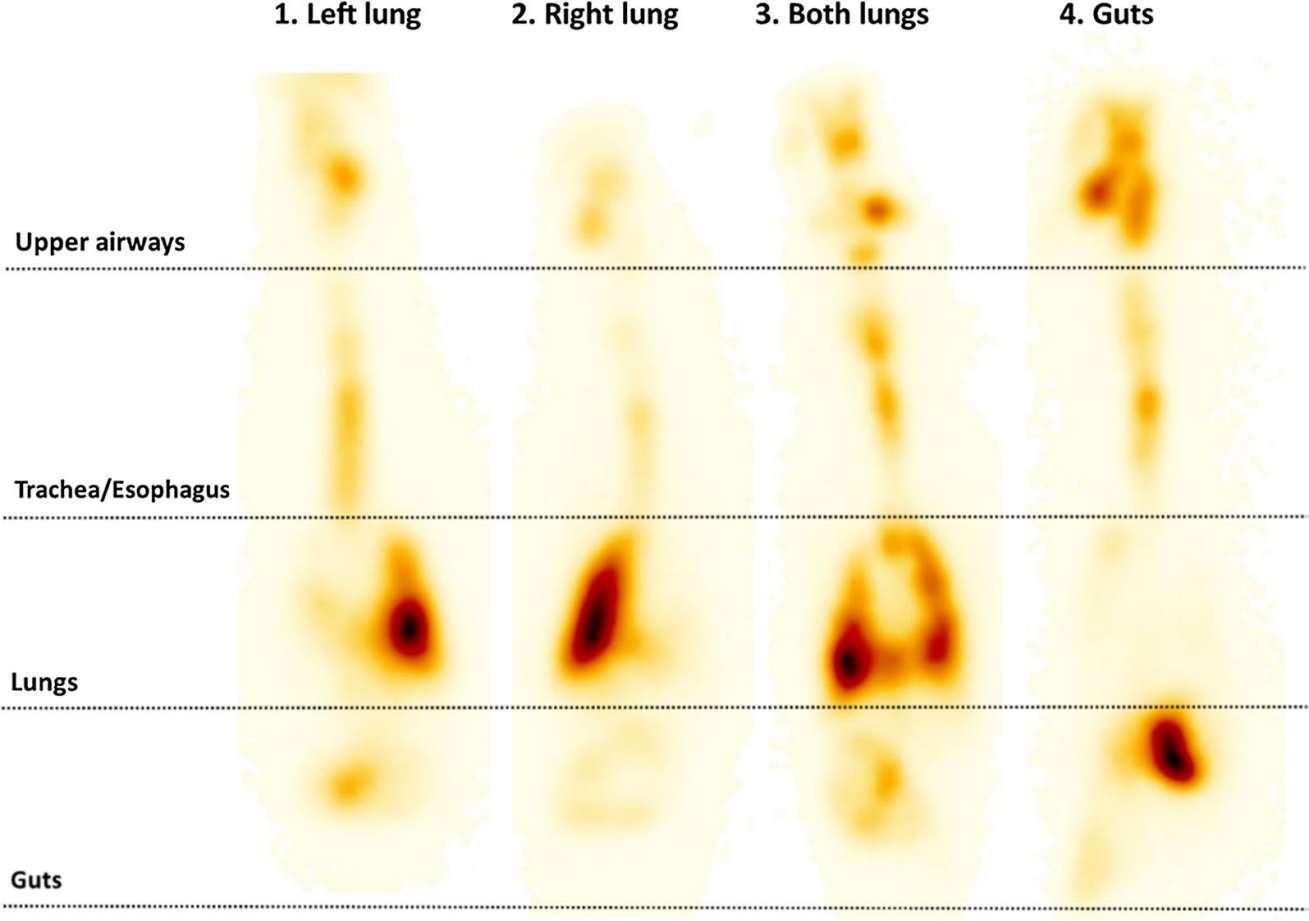
Gamma scintigraphy of newborn piglets obtained after nebulization of 99 m-technetium (^99m^Tc)-labelled surfactant. These images belong to the recent study conducted by Cunha-Goncalves et al*.* [130]

Although the study by Dubus et al. (mentioned in Table 5) was not conducted in spontaneously-breathing animals, this investigation was among the first studies demonstrating the significantly superior performance of the vibrating-membrane technology (Aeroneb Pro) compared with a low-flow jet nebulizer (MistyNeb) in the context of neonatal nebulization (Table 5) [77]. Linner et al*.* used an investigational vibrating-membrane nebulizer with eFlow technology to deliver surfactant aerosols to newborn piglets managed with CPAP, which was delivered either using a mask or nasal prongs as the animal interface [122]. The median lung deposition observed using mask or prongs was 5% and 14% of the nominal dose, respectively, although they report a high inter-animal variability in lung deposition. A recent study by the same group indicates a slightly superior mean lung deposition of surfactant when the NIV support was nIPPV (21.6%) compared to nCPAP (15.9%) [137]. On the contrary, lung deposition of drugs during HFNC ventilation is very low (< 1%) irrespective of using jet or vibrating-membrane nebulizers [93]. Besides lung deposition, these studies also permitted determination of drug deposition in the nose, trachea, gut, nebulizer, and NIV circuit. Most importantly, the pulmonary distribution of the nebulized drug can be analysed. Figure 7 shows an example of gamma scintigraphy images of different piglets after inhalation of radiolabelled surfactant via nasal prongs [130].

Figure 7 illustrates the variability in terms of both radiolabelled surfactant lung deposition and distribution. For instance, animals 1 and 2 were positioned in lateral decubitus during nebulization of *Poractant alfa.* However, animal 1 was positioned with the right side up and animal 2 with the left side up. Interestingly, even though total lung deposition was 38.6% and 47.8% in animal 1 and 2, respectively, surfactant deposition in the dependent lungs accounted for 33.4% and 43.7% in each case, suggesting the formation of a surfactant film within the airways after nebulization which distributes preferentially to the dependent lung [122, 130]. Nevertheless, animal 2 and animal 4 were both positioned with the left side up but the difference in lung deposition was evident: total lung deposition in animal 4 was just 3.3%, whereas deposition in the nasopharynx (18.4%) and stomach (20.6%) accounted for 39% of the nominal surfactant dose. Animal 3 was positioned in prone position and achieved a bilateral surfactant lung deposition that accounted for a 39.6% of the nominal dose (left lung 18.1%; right lung 21.5%). In a clinical setting, an uneven surfactant distribution as shown in Fig. 7 may induce a transient lung improvement but may ultimately yield a poor clinical response to therapy [175], such as unilateral pulmonary interstitial emphysema. Therefore, adequately powered lung deposition and distribution studies are essential in the development of aerosol therapies intended for the treatment of spontaneously-breathing premature infants managed with NIV. Nevertheless, such lung deposition and distribution studies also have a number of limitations that must be considered. Firstly, there is a difference between the nasal anatomy of animals and humans. For instance, if we consider neonatal piglets, the upper airway deposition pattern may be slightly different compared to that of human preterm neonates. Further, the piglets may also require custom made animal interfaces. Finally, one should also bear in mind that lung distribution studies like the ones presented in Table 5 are conducted with healthy animals and therefore do not capture the pathophysiological features of lung diseases (e.g. atelectasis, reduced *V*_T_, poor compliance…).

#### In vitro models

The use of in vitro models to investigate aerosol deposition in the context of neonatal drug delivery has gained momentum over recent years. This approach is very useful to decipher the influence of a great number of technical variables that affect the overall aerosol delivery efficiency before entering preclinical studies or clinical trials. Conventional in vitro tests are aimed at determining the aerosol production rate of specific device/product combinations as well as the particle size distribution of the aerosol plume [176, 177]. These preliminary tests are particularly relevant for medical aerosols of complex formulations of higher viscosity than aqueous solutions that additionally display surface activity (e.g. surfactants and liposomal formulations) because they significantly affect the output rate of the nebulizers and may yield different particle size distributions [83, 121, 178–180]. The aerosol output rate is usually determined by collecting the aerosol emitted by the aerosol generator in specific drug-collection filters placed directly after the nebulizer [105, 111]. The particle size distribution can be determined using different techniques and devices such as the Next Generation Impactor (NGI) [181, 182], Andersen Cascade Impactor (ACI) [183], Laser Diffraction [184], or Time of Flight (TOF) [177] technologies. The theoretical principles of these methods were reviewed by Mitchell and Nagel [185].

**Fig. 8:**
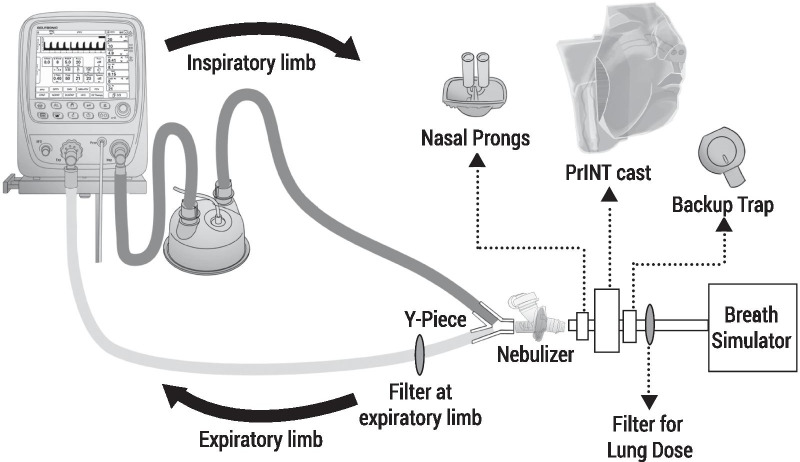
Scheme of an in vitro neonatal circuit for aerosol delivery experiments. The set-up is composed of a neonatal ventilator to provide NIV support followed by a temperature and humidity control unit, a nebulizer placed immediately after the Y-piece, a patient interface (nasal prongs), a cast of the upper airways of a premature neonate (PrINT model), a backup trap to collect the aerosol that impacts in the cast and moves forward as a liquid, drug collecting filters to determine the lung dose and the amount of aerosol reaching the expiratory limb, and a breath simulator programmed with a sinusoidal breathing pattern of a premature neonate. adapted from [182, 184]

The aforementioned United States Pharmacopeia’s chapter < 1601 > *Products for Nebulization-Characterization Test* provides a clear guideline to characterize the amount of drug delivered as well as the aerodynamic assessment of the aerosols generated by a given drug/nebulizer combination [90]. This method provides robust data on the emitted aerosol dose and its particle size distribution. Unfortunately, it does not provide a good estimate of the lung dose, particularly in the context of aerosol delivery to spontaneously-breathing preterm infants due to non-consideration of the respiratory pattern of preterm neonates, the NIV configuration, its bias flow, or the small size of the upper airways. More sophisticated in vitro ventilation circuits were developed to circumvent these limitations. Figure 8 shows a representative scheme of an in vitro neonatal NIV circuit designed for aerosol deposition studies [184]. Such in vitro set-ups typically include NICU-approved NIV generator devices (e.g. neonatal ventilator, bubble CPAP device, Infant Flow®) with appropriate tubing, humidity and temperature control, a breath simulator programmed with the breathing pattern approaching that of premature neonates, and drug collection filters to determine the lung dose. Notably, several neonatal in vitro circuits also include casts of the upper airways of neonates derived from three-dimensional computed tomography (CT) or magnetic resonance imaging of human patients, which can be digitalized and further translated into hard-copies by rapid prototyping [76, 93, 105, 109]. Considering that newborn infants are preferential nasal breathers, nose-throat casts accurately mimicking the anatomy and the dimensions of the upper airways are also very useful for aerosol deposition studies. Janssens et al*.* developed the so-called Sophia Anatomical Infant Nose-throat (SAINT) model, which was derived from the CT scan of a 9-month-old girl of 10 kg [183]. Although the SAINT cast has been used as a model for premature neonates [93, 186], its dimensions are markedly larger than those of these patients. Using similar approaches, Minnochieri et al*.* developed a Premature Infant Nose Throat-Model (PrINT) from a 32-week gestational age infant of 1.75 kg [187] and Younquist et al*.* generated a model from a head CT scan from a 26-week gestational age infant [188].

The relevance of incorporating a cast of the upper airway in the circuit was highlighted recently by Bianco et al*.* [182]*.* In this study, the authors assembled two different NIV ventilation circuits to determine the delivered dose of nebulized surfactant. The first set-up was designed based upon the United States Pharmacopeia guidelines and was composed of a neonatal ventilator (air-flow 5 L/min, to achieve 5 cmH_2_O of CPAP), an eFlow Neos vibrating-membrane nebulizer, nasal prongs, a drug collection filter placed immediately after the prongs, and a breath simulator with the breathing pattern of a premature neonate (*V*_T_ = 4.85 mL/kg; respiratory rate = 70 cycles/min; I:E = 1:1.5). In the second set-up, in addition to all the aforementioned elements, the PrINT cast [187] and a liquid-collecting trap were sequentially placed between the nasal prongs and the drug collection filter (Fig. 8). The liquid-collecting trap was placed between the PrINT cast and the drug collecting filter to drain the amount of nebulized surfactant impacting against the inner walls of the prongs and the PrINT cast that would otherwise move towards the filter as a liquid film. The amount of surfactant collected in the drug filter with the first set-up ranged between 63 and 75% of the nominal dose (1056 mg of *Poractant alfa*), whereas it was remarkably reduced to values ranging 10–20% in the second set-up, which might be considered to be more representative of the in vivo situation. Interestingly, one-third of the nominal dose was recovered within the liquid-collecting trap, which highlights the significant aerosol impaction that takes place in the upper airways under conditions more representative of the neonate.

Therefore, advanced neonatal NIV circuits provide a controlled framework to carry out more insightful aerosol deposition studies. These types of neonatal in vitro models have been used to investigate the aerosol delivery efficiency using different NIV types [109], to compare the performance of jet and vibrating-membrane nebulizers [76, 94, 121], the effect of the bias flow on aerosol deposition [94, 187], the influence of nebulizers positioning in different locations of the circuit [94, 184], the feasibility of using different NIV interfaces [182], and to estimate the lung dose in dose-escalating studies [184]. Nevertheless, the “model” concept implies a simplification of reality and thus gives rise to a number of limitations. Firstly, the inhaled dose is usually determined by measuring the amount of drug impacting and remaining in a drug collection filter placed in the distal outlet of the upper airway cast, which precludes that a fraction of the inhaled aerosol could be exhaled during the expiratory phase. Secondly, the oral and nasal air-leaks that typically occur during NIV in the NICU are not considered in these models. Generally, all components of in vitro neonatal circuits are tightly assembled to avoid air leaks and nose-throat casts (e.g. PrINT and SAINT models) do not consider the oral cavity, which may often act as a relieve valve during NIV in spontaneously-breathing infants. Assuming that medical aerosols are distributed homogeneously in the inspiratory air-flow, air-leaks may proportionally reduce the inhaled dose. Another limitation refers to the breathing pattern of premature neonates used in in vitro studies; breath simulation in bench studies is typically mimicked by programming a fixed sinusoidal pattern and therefore does not capture the complex respiratory patterns of premature neonates, which may include apnoeic episodes and irregular periodic breathing patterns. Consequently, in vitro studies may slightly overestimate the lung dose. Lastly, the regional lung distribution of aerosolized drugs cannot be investigated with in vitro models. Interestingly, however, Montigaud et al*.* recently described an ex vivo model of BPD consisting of the 3D-printed SAINT model connected to a sealed enclosure containing a rabbit thorax [186]. In this model, rabbit lungs were ventilated with BPD breathing patterns generated by negative pressure; lung ventilation assessment was performed with ^81m^Krypton scintigraphy and regional aerosol deposition was determined by coupling ^99m^Tc-DTPA.

As a final remark, the use of computational fluid dynamic (CFD) simulations, also referred to as in silico studies, has great potential as a tool to improve neonatal aerosol delivery [189, 190], although the technique remains largely under-exploited in this area so far. CFD simulations have significant and broad potential applicability and may eventually be used to study aerosol formation (e.g. liquid breakup or powder degradation), to evaluate and improve the aerosol generator device performance [191], to aid the design of circuits and devices (e.g. patient interfaces) aimed at reducing extra-thoracic deposition of aerosols [78, 118], and to investigate the regional airway deposition of new aerosol delivery strategies [192].

## Conclusions and perspective

Major advances in the treatment of neonatal lung disease have been made in recent decades to increase survival through greater use of NIV to avoid the often-detrimental pro-inflammatory consequences associated with the use of mechanical ventilation. The shift in clinical practice toward NIV has led in turn to an increased focus on non-invasive delivery of drugs to target the lungs, such as surfactants. However, the knowledge gained on aerosol therapy in the paediatric population cannot be translated directly to the neonates, in particular when considering preterm infants.

None of the attempts to deliver surfactant as an aerosol have yet proven to be clearly efficacious in the clinical studies published to date, indicating the challenge of targeted lung delivery in this patient population and the lack of comprehensive understanding of the intricate combination of factors impacting this route of administration. The authors have described all limiting factors that can affect lung deposition, which ultimately negatively impact the therapeutic benefit.

For example, and notably, all drugs approved for nebulization in the paediatric population are to be administered via facial masks. This approach is potentially problematic for the preterm neonate, who are preferential nasal breathers, unless the mouth is kept closed during aerosolization, similar to the approach by Minocchieri [73]. The authors would discourage the development of aerosolized drugs to be administered exclusively via the mouth, bypassing the nasopharynx, since this method approaches pharyngeal instillation and may further reduce the dose delivered to the lungs.

Different NIV strategies rely on different patient interfaces, which we have shown can play a significant role in the amount of drug that can be inhaled by the patients. For instance, HFNC can be applied safely only with dedicated cannulas and a high degree of leakage; hence, nebulizing a drug in combination with HFNC should be discouraged. Other NIV techniques can be applied with both nasal prongs and masks and there is insufficient evidence to recommend one versus the other. Irrespective of this choice, what is likely most important is the degree of leakage that is difficult to control and to be kept at minimal levels without impairing the safety of the procedure.

The position of the nebulizer in the respiratory circuit clearly has an effect on the inhaled dose: nebulizers should not be placed in the inspiratory limb as there is sufficient evidence that lung deposition is negligible with this technique. Similarly, jet nebulizers should also be avoided, as they produce high velocity particles and if positioned at the patient interface such devices would create an aerosol that would impact more rapidly on the surrounding surfaces (both the device interface and the patient’s mucosa).

Unlike in adults, radiolabelling an active ingredient and tracing its deposition in the lungs is not possible in the neonatal population for safety reasons. Thus, assumptions about the lung dose and distribution in neonates are reliant on in vitro and animal models; however, inappropriate model selection may overestimate the delivered lung dose. The amount of drug that should be administered to attain a therapeutic dose in the lung could be several folds higher than expected due to leakage at the patient interface, with potential implications for cost-effectiveness.

The lack of clear knowledge of the requirements for the development of aerosolized drug from premature infants is mirrored by the lack of guidelines from regulatory authorities and pharmacopeia on the drug development of such treatment. In conclusion, we believe that further studies are needed to understand if there is a solution that addresses all factors (both intrinsic and extrinsic) influencing drug aerosolization in combination with NIV and which would support optimized and higher lung deposition and in turn convey a meaningful therapeutic benefit. This would allow the leveraging and deployment of what is, in principle, a very attractive non-invasive drug delivery modality. To do so, a strong collaboration between academia, industry, and regulatory bodies is needed.

## Data Availability

Not applicable.

## Abbreviations

^99m^Tc-DTPA: ^99m^Tecnetium Diethylenetriamine Pentaacetate
ACI: Andersen cascade impactor
APSD: Aerodynamic particle size distribution
BiPAP: Bilevel positive airway pressure
BPD: Bronchopulmonary dysplasia
CAG: Capillary aerosol generator
C_dyn_: Dynamic compliance
CFD: Computational fluid dynamic
COPD: Chronic obstructive pulmonary disease
CPA: Continuous powder aerosolizer
CPAP: Continuous positive airway pressure
CT: Computed tomography
DPI: Dry powder inhalers
ECG: Enhanced condensational growth
FPF: Fine particle fraction
FRC: Functional residual capacity
G: Generation
GA: Gestational age
GC: Graseby capsule
GSD: Geometric standard deviation
HFNC: High flow nasal cannula
I:E: Inspiratory-expiratory ratio
ID: Internal diameter
IQR: Interquartile range
MMAD: Mass median aerodynamic diameter
MMD: Mass median diameter
NA: Not available
NAVA: Neurally adjusted ventilator assist
nCPAP: Nasal continuous positive airway pressure
NGI: Next generation impactor
NICU: Neonatal intensive care unit
nIPPV: Nasal intermittent positive pressure ventilation
NIV: Non-invasive ventilation
nRDS: Neonatal respiratory distress syndrome
PEEP: Positive end expiratory pressure
pMDI: Pressurized metered dose inhalers
PrINT: Premature infant nose throat-model
PSD: Particle size distribution
SAINT: Sophia anatomical infant nose-throat
sNIPPV: Synchronized nasal intermittent positive pressure ventilation
T_i_: Inspiratory time
TOF: Time of flight
*V*_E_: Minute volume
VMD: Volume median diameter
*V*_T_: Tidal volume
